# Coumarin-Mediated Inhibition of Diadenylate Cyclase Correlates with Impaired Biofilm Formation in *Streptococcus mutans*

**DOI:** 10.1021/acsinfecdis.5c00767

**Published:** 2026-07-01

**Authors:** Edwin M. Rojas, Abhishek Govindan, Parvathy Babu, Soniya Joseph, Hua Zhang, Yanting Zhu, Tyrese Boddie, Hui-Ting Lee, Hui Wu, Sadanandan E. Velu

**Affiliations:** † Department of Chemistry, 9968 University of Alabama at Birmingham, Birmingham, Alabama 35294, United States; ‡ School of Dentistry, University of Alabama at Birmingham, Birmingham, Alabama 35294, United States; § Global Center for Craniofacial Oral and Dental Disorders, University of Alabama at Birmingham, Birmingham, Alabama 35294, United States; ∥ Center for Clinical and Translational Sciences, University of Alabama at Birmingham, Birmingham, Alabama 35294, United States; ⊥ Department of Biomaterial and Biomedical Sciences, School of Dentistry, 6684 Oregon Health & Science University, Portland, Oregon 97239, United States

**Keywords:** S. mutans, diadenylate cyclase, c-di-AMP, biofilm, inhibitor, coumarin

## Abstract

*Streptococcus mutans* diadenylate cyclase (*Sm*DAC) catalyzes the cyclization of two ATP molecules into cyclic di-AMP, a second messenger that regulates many cellular processes including biofilm formation. Aided by structure-based drug design and subsequent structure–activity relationship studies, we identified a coumarin chemotype as low-micromolar inhibitors of *Sm*DAC. Optimized lead compounds inhibited both biofilm formation and planktonic growth of *S. mutans*. Biofilm inhibition marginally exceeded growth inhibition at tested doses, indicating relative selectivity toward biofilm inhibition. Additionally, the lead inhibitor did not significantly affect the growth and biofilm of representative commensal streptococci in a mixed-species community consisting of *S. mutans*, *S. gordonii*, and *S. sanguinis* at 10 μM, though this selectivity was lower when tested in single-species growth and biofilm conditions. Overall, this study demonstrates that the inhibition of *S. mutans*’ DAC and biofilm by small molecules is a potential strategy for the treatment and prevention of dental caries.

## Graphical abstract

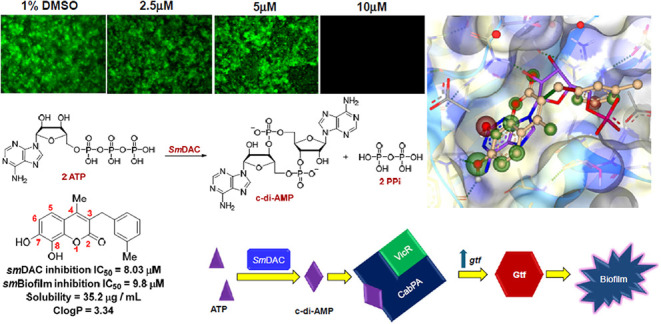

## Introduction

Dental caries is one of the most prevalent infectious diseases that affects human oral health.^1^ Dental plaque comprises of a complex microbiome of more than 700 microorganisms, several of which possess virulence factors and defense mechanisms that aid robust biofilm formation under disease conditions.^2^ *Streptococcus mutans*, a Gram-positive bacterium, is recognized as the primary etiologic agent for the development of dental caries.^3,4^ Current dental caries treatments have important limitations. Rapid recolonization of the oral bacteria renders conventional hygiene practices such as toothbrushing or mouthwashes less effective.^5^ The nonselective antimicrobial agents commonly used in mouthwashes affect both pathogenic and beneficial commensals, leading to adverse effects.^6^ In addition, cariogenic bacteria resist traditional antimicrobial treatments by forming and thriving in biofilms.^7^ Preventive and therapeutic strategies to address this challenge by targeting different virulence factors of *S. mutans* are under investigation.^8–10^ Although prior work targeting *S. mutans* glucosyl transferases (Gtfs) to inhibit glucan synthesis has shown promise in *in vitro* and *in vivo* studies,^11–17^ no treatment has yet reached clinic.

In the absence of an effective clinical preventative approach to inhibit cariogenic biofilms, there remains an unmet need to discover biofilm inhibitors which act by novel mechanistic pathways.^18^ *S. mutans* diadenylate cyclase (*Sm*DAC) is an intracellular enzyme that can be leveraged for the design and discovery of biofilm inhibitors.^19–23^ *Sm*DAC, in the presence^24^ of Mn^2+^ converts two ATP molecules into cyclic di-AMP (c-di-AMP), a second messenger that regulates many cellular processes.^25^ *Sm*DAC belongs to the CDA family of DAC enzymes that exhibit optimal enzymatic activity with Mn^2+^ over Co^2+^, as reported previously.^24^ In *S. mutans*, DAC enzyme promotes biofilm formation by intracellular binding of c-di-AMP to CabPA/VicR complex, which results in the upregulation of the *gtf* genes.^22^ Thus, developing *Sm*DAC inhibitors is a promising strategy to inhibit cariogenic biofilms. Our prior work in this area has identified (+)-brazilin as a noncompetitive *Sm*DAC inhibitor with antibacterial activity.^24^ Here, through high-throughput *in silico* screening, we identified a novel *Sm*DAC inhibitor STL372167 (Figure 1B) and established its enzyme inhibitory and antibacterial activities across oral streptococci. STL372167 features a coumarin core, a pharmacophore with broad therapeutic relevance.^26–29^

**Figure 1:**
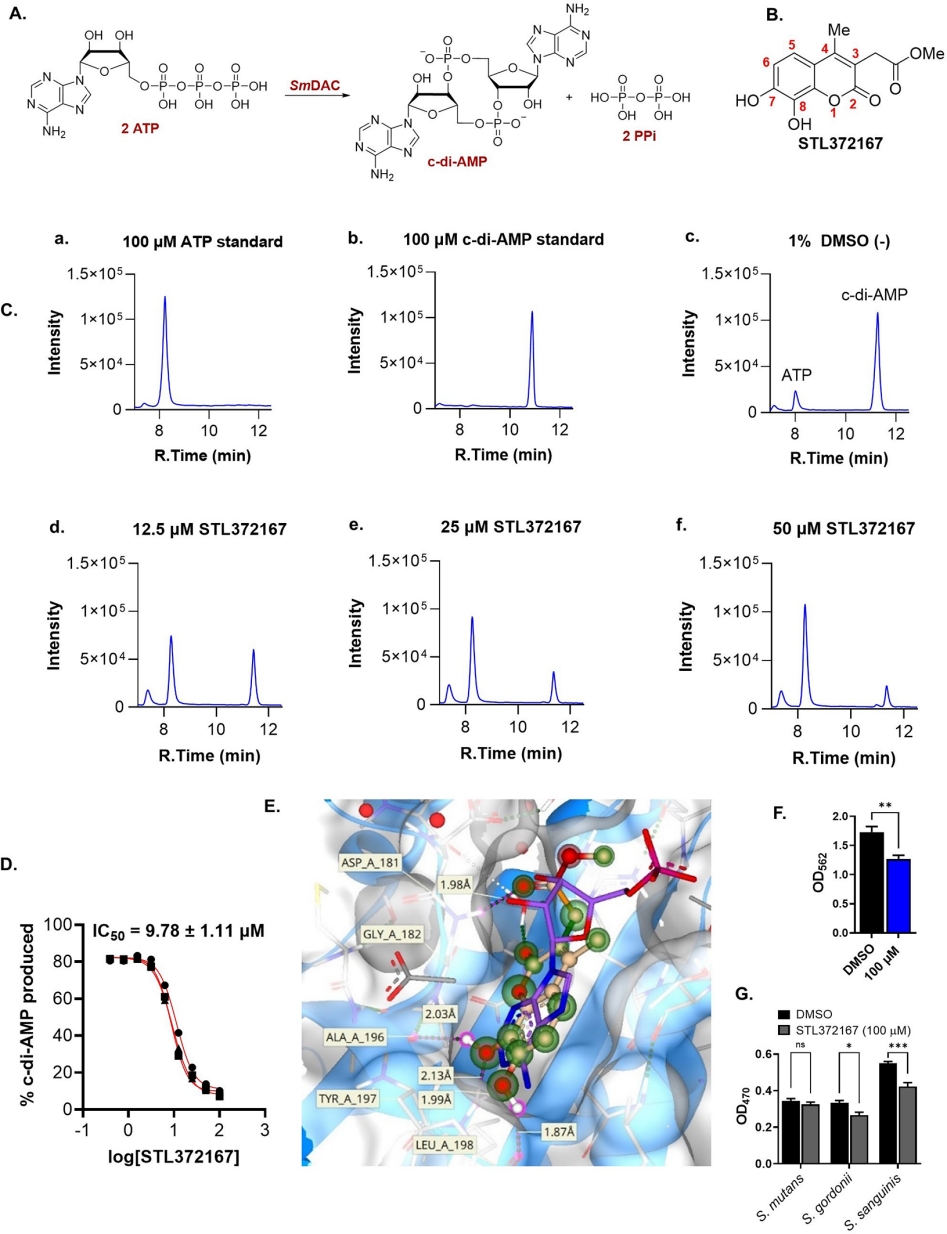
**A)**Scheme of catalytic reaction of *Sm*DAC that converts two ATP molecules to cyclic-di-AMP + 2PPi. **B)** Chemical structure of STL372167. **C)** HPLC chromatograms from *Sm*DAC inhibition assay used to quantify the percentage conversion of ATP to c-di-AMP. **a)** ATP standard. **b)** c-di-AMP standard. **c)** Enzymatic reaction treated with 1% DMSO as negative control. **d)** Enzymatic reaction treated with 12.5 μM of STL372167. **e)** Enzymatic reaction treated with 25 μM of STL372167. **f)** Enzymatic reaction treated with 50 μM of STL372167. **D)** STL372167 inhibits c-di-AMP production in a dose-dependent manner in the HPLC assay. **E)** Docking model of STL372167 (wheat sticks) in the AMP (purple sticks) binding site of *Sm*DAC/AMP crystal structure generated using SeeSAR 13.0.1. Green HYDE coronas indicate atoms with favorable interactions and red HYDE coronas indicate unfavorable interactions. **F)** *S. mutans* UA159 treated with 100 μM of STL372167; biofilm biomass measured at OD_562_ using the crystal violet assay. **G)** *S. mutans* UA159, *S. gordonii*DL1, and *S. sanguinis* SK36 treated with 100 μM of STL372167; planktonic growth measured at OD_470_. Each of the biofilm and growth assays was conducted in triplicate. Statistical significance was determined with one-way ANOVA. ns = *p* ≥ 0.05, * = *p* < 0.05, ** = *p* < 0.01, and *** = *p* < 0.001.

Coumarins have been reported to have antibacterial,^30^ antiviral,^31^ antifungal,^32^ anti-inflammatory,^33^ and anticancer^34^ activities. Antibacterial effects of coumarins toward Gram-positive bacteria, including *S. mutans* and Gram-negative bacteria have been reported.^35^ Bactericidal and bacteriostatic activity against *S. mutans* has been reported for *Mammea americana* extract, the major metabolite of which is a coumarin.^36,37^ Coumarin-N-heterocyclic hybrids designed to target topoisomerase II were found to have antibacterial activity against *S. mutans*, *S. aureus*, *P. aeruginosa*, and *E. coli* with submicromolar minimum inhibitory concentration (MIC) values. Bis-coumarin derivatives have shown antibacterial activity against *S. mutans* by inhibiting DNA gyrase and topoisomerase IV.^38^ The coumarin antibiotic novobiocin targets DNA supercoiling enzyme DNA gyrase B in bacteria for its antibacterial activity.^39,40^ In addition, inhibitory activity of coumarins against *P. gingivalis* biofilm is caused by the reduction in the quorum sensing (QS) activities or by the downregulation of biofilm-related regulatory genes such as *vimA, rgPA*, *luxS*, and *mfa1*.^41^ Additionally, coumarins designed to act upon enzymes FabI and FabK resulting in activity against bacterial growth and survival.^42,43^ Furthermore, coumarins have also been reported to inhibit FtsZ, which plays an important role in bacterial cell division.^44^ Herein, we report for the first time, a novel mechanism by which coumarins inhibit *S. mutans* biofilm and growth by targeting the c-di-AMP production by *Sm*DAC.

## Results and Discussion

### *In Silico* Screening and Lead Identification

We screened a 1.1 M compound library against the AMP binding site of the *Sm*DAC/AMP crystal structure (PDB code: 7L8N) using FlexX 6.0, within SeeSAR 13.0.1 ^45^ on the KNIME 4.7.3 analytical platform^46^ to identify 66 candidate small-molecule compounds as potential *Sm*DAC inhibitors. All 66 compounds were evaluated in a *Sm*DAC inhibition assay reported previously from our lab.^24^ This assay quantifies c-di-AMP produced by the enzymatic activity of *Sm*DAC on ATP (Figure 1A). Inhibition of c-di-AMP production by the compounds is determined by quantifying the peak areas of c-di-AMP in the HPLC chromatograms before and after the compound treatment (Figure 1Ca-f). When tested at 100 μM in the HPLC enzymatic assay, STL372167 was found to be the most potent compound, inhibiting *Sm*DAC by 86.7%. STL372167 showed dose-dependent inhibition of the conversion of ATP to c-di-AMP with an IC_50_ value of 9.78 μM (Figure 1D).

Our docking model suggests that the coumarin ring of STL372167 aligns with the adenine ring in the AMP in the hydrophobic region of the active site suggesting that it could be a competitive inhibitor. A key structural feature required for the enzymatic activity in all DACs is the triad, Asp-Gly-Ala found in the loop between α-helix 3 and β-sheet 4.^19^ As predicted by the model, the ability of STL372167 to form strong H-bonds with the residues in the triad, Asp181, Gly182, and Ala196, further supports its mechanism of inhibition of *Sm*DAC. In addition, Tyr197 is also an essential residue in stabilizing AMP through π–π stacking interactions with the adenine ring.^47^ Additionally, the aromatic ring of STL372167 was found to stack with Tyr197 in our model (Figure 1E).

The ADME properties of STL372167 predicted by Optibrium in SeeSAR 13.0.1 corresponds to favorable drug-like properties with good ligand efficiency, minimal torsional strain, minimal intra- and intermolecular clashes, a TPSA of 93.1, a CLogP of 1.35, and estimated binding affinity within nM−μM range. HYdrogen bond and DEhydration (HYDE) calculations in SeeSAR 13.0.1 showed that STL372167 has favorable interactions within the active site represented by 13 green coronas with only one unfavorable red corona in the docking model. The 7-OH group of the coumarin forms H-bonds with the CO group (1.87 Å) of Leu198 and the NH group (1.99 Å) between Tyr197 and Leu198. The 8-OH group forms H-bonds with the CO group (2.03 Å) of Ala196 and the NH group (2.13 Å) between Tyr197 and Leu198. The CO of the ester moiety at the 3-position forms H-bonds with the NH group (1.98 Å) between Asp181 and Gly182 (Figure 1E).

STL372167 was then evaluated in an *S. mutans* single-species biofilm assay,^17,48^ which showed only a modest 30% inhibition at 100 μM (Figure 1F). Despite being a potent *Sm*DAC inhibitor, the observed modest biofilm inhibition can be attributed to its low lipophilicity and associated poor cell membrane permeability as indicated by its low CLogP value of 1.35 (Table 1). Therefore, one of the goals of this study was to improve the lipophilicity of this molecule through SAR studies. To examine the selectivity toward biofilm formation versus bacterial growth, the bactericidal effects of STL372167 against *S. mutans* were evaluated. The compound treatment at 100 μM did not significantly alter the growth of *S. mutans* growth compared to the control group (Figure 1G), suggesting that the observed biofilm inhibition at this treatment dose is not due to bactericidal activity. STL372167 was also evaluated for its ability to inhibit two oral commensal streptococcal species, *S. gordonii* and *S. sanguinis* at 100 μM. Unlike *S. mutans*, STL372167 inhibited the cell viability of *S. gordonii* by 20% and *S. sanguinis* by 25% (Figure 1G), suggesting that the compound could be bactericidal to commensal streptococci at this dose.

**Table 1:**
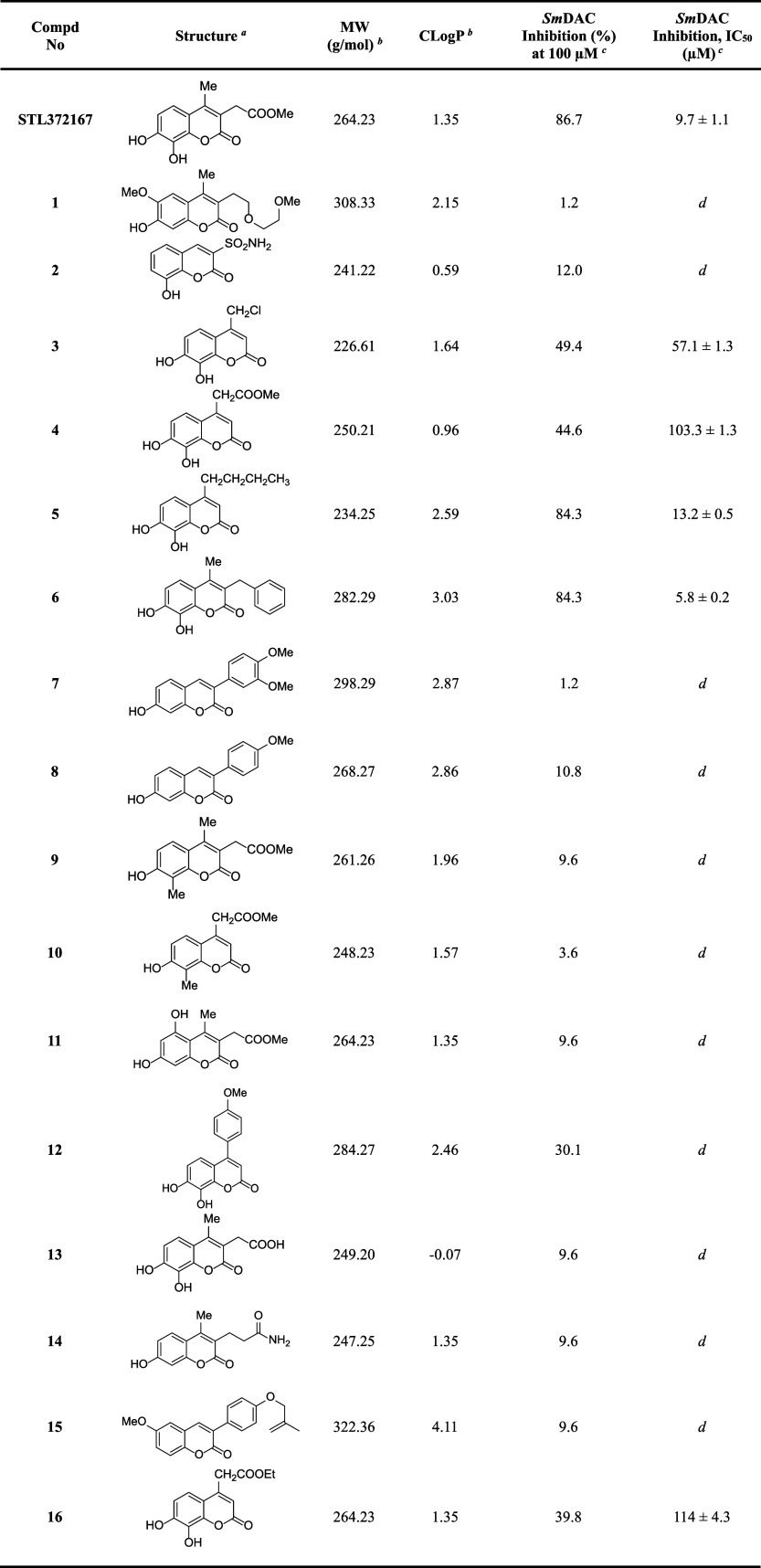
Results of *Sm*DAC Enzymatic HPLC Assay and Drug Properties of 16 Coumarins Obtained from Molport^a^ ^b^ ^c^ ^d^

a Coumarins obtained from Molport.

b Calculated using Optibrium in SeeSAR 13.0.1.^45^

c Determined by the HPLC enzymatic assay quantifying the conversion of ATP to c-di-AMP by SmDAC,^24^

d Not determined as they were relatively weak inhibitors.

### Similarity Search

The next step in this study was to improve the biofilm inhibition activity of STL372167 by rational drug design approaches. In this regard, we first conducted a query in Molport to identify commercially available coumarins similar to STL372167. One of the goals of this search was also to identify molecules with improved lipophilicity. The 105 compounds identified from this search were docked in the *Sm*DAC/AMP binding site and 16 potential leads were selected based on their estimated binding affinities, structural diversity, and synthetic feasibility (Table 1).

The *Sm*DAC inhibition activities of 16 coumarins along with STL372167 at 100 μM were determined (Table 1). Analogs **5** and **6** showed the strongest activity with 84.3% inhibition. Analogs **3**, **4**, and **16** displayed moderate activity with 39–49% inhibition. Others were weak inhibitors. Though the tested compounds were not systematically designed for SAR analysis, multiple structure–activity relationships were evident in the enzymatic assay results. Moving the polar CH_2_COOCH_3_ group at position-3 of STL372167 to position-4 substituting the methyl group as in **4** resulted in 50% reduction in activity, suggesting the need for the side chain at position-3 and the need of hydrophobic group at position-4 for the activity. The modification of the CH_2_COOCH_3_ group at position-4 to CH_2_COOCH_2_CH_3_ group in **16** further decreased the activity, suggesting that an increase in length of this group past four atoms is not favorable. In addition, having a hydrophobic butyl group at position-4 in **5** versus a polar CH_2_COOCH_2_CH_3_ group in **16** favors *Sm*DAC inhibition as the butyl group likely interacts with the hydrophobic residues in the active site.

Analysis of the structures of the weak inhibitors also revealed certain structural features that are unfavorable for the enzyme inhibition. Replacing the ester group at position-3 of STL372167 with a carboxylic acid in **13** drastically reduced the activity from 86.7% to 9.6%, suggesting less hydrophilicity at this position favors the activity. Removal of 8-OH or its replacement with a methyl group has significantly reduced activity in several analogs. For example, replacing the 8-OH of STL372167 with a methyl group in **9** caused a drastic reduction in activity from 86.7% to 9.6%. A similar effect was observed when the 8-OH in **4** was replaced with a methyl group in **10** decreasing the activity from 44.6% to 3.6%. Supporting this hypothesis, it was found that several less active compounds such as **1**, **7**, **8**, **10**, **11**, **14**, and **15** did not contain an 8-OH group, which emphasizes the importance of H-bonding of 8-OH with the CO group of Ala196 and the NH group between Tyr197 and Leu198.

Compound **6** identified from this screen was selected for further analysis due to its potent *Sm*DAC inhibition with an IC_50_ value of 5.8 μM, improved lipophilicity as indicated by its CLogP value of 3.03, and the solubility of 15.7 μg/mL. Compound **6** displayed considerably better biofilm inhibition than STL372167 with an IC_50_ value of 42.8 μM (Figure 2A), suggesting that it is due to improved enzymatic activity and lipophilicity of **6**. SYTO9 staining bacterial cells within biofilms showed significant reduction at 40 μM and almost complete disappearance at 80 μM (Figure 2B, panel I). In addition, presence of glucans detected by staining using Cascade Blue-dextran conjugated dye, was also significantly reduced at 40 μM and almost completely disappeared at 80 μM (Figure 2B, panel II). Additionally, the presence of eDNA in biofilms was detected using propidium iodide, which showed a noticeable reduction of eDNA at 40 μM and almost complete disappearance at 80 μM (Figure 2B, panel III).

**Figure 2:**
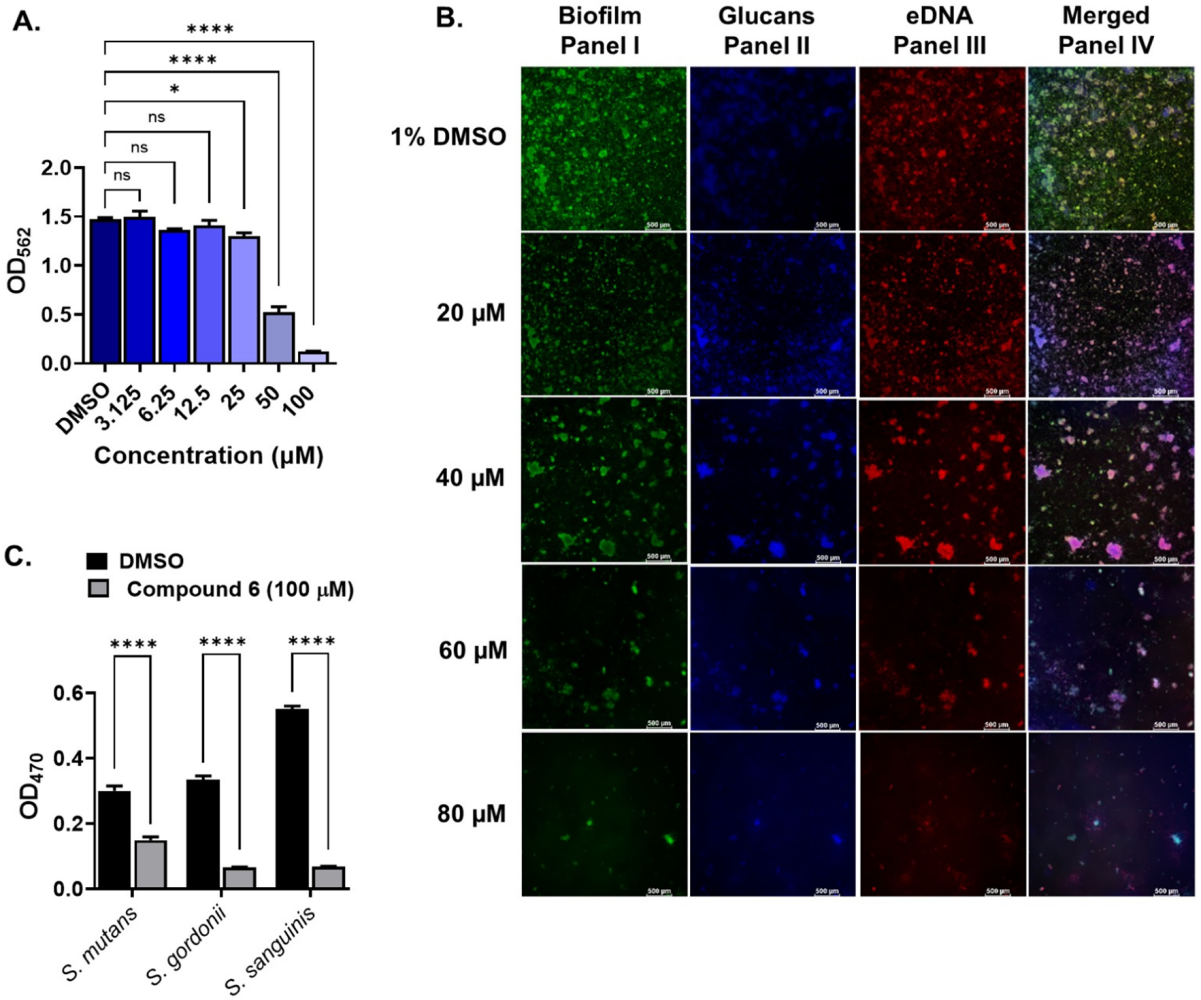
**A)** *S. mutans* UA159 cells were treated with compound **6** at the indicated concentrations and the biofilm biomass was quantified at OD_562_ in the crystal violet assay. **B)** Representative fluorescence micrographs of UA159 biofilms after 16 h of treatment with the indicated concentrations of compound **6**. Bacterial cells stained with SYTO9 (green, panel I); glucans stained with Cascade Blue–dextran conjugated dye (blue, panel II); eDNA stained with propidium iodide (red, panel III); and merged micrographs (panel IV). **C)** *S. mutans* UA159, *S. gordonii* DL1, or *S. sanguinis* SK36 were treated with compound **6** at 100 μM and planktonic growth was measured at OD_470_. Data for biofilm and planktonic growth are from three independent experiments. Statistical significance was tested with one-way ANOVA. ns = *p* ≥ 0.05, * = *p* < 0.05, and **** = *p* < 0.0001.

Compound **6** did inhibit the growth of *S. mutans* by 50% at 100 μM (Figure 2C). However, its ability to inhibit the biofilm at the same dose was about 95%, indicating selectivity toward biofilm inhibition at this dose. Compound **6** was also found to be bactericidal to the commensal streptococci *S. gordonii* and *S. sanguinis* by inhibiting their growth by 80% at 100 μM treatment dose (Figure 2C).

### Structure–Activity Relationship and Lead Optimization Studies

Based on the improved IC_50_ values for the *Sm*DAC inhibition, biofilm inhibition and enhanced lipophilicity, the benzyl derivative, **6** was selected for further SAR and design of eight analogs to further glean on the positions and functional groups of the molecule that are important for its activity (Figure 3). In this group of compounds, we explored the effect of substituents on the benzyl ring of **6** as well as its removal (**22**) and replacement with COOH (**23**) and COOMe (**24**) groups. Among the substitutions on the benzyl ring, we explored the effects of electron withdrawing groups such as 3-CF_3_ (**17**), 4-CF_3_ (**18**), 4-Cl (**19**), 4-F (**20**), and electron donating group 3-Me (**21**) group. In addition to exploring the electronic effects, such substitutions are also meant to increase lipophilicity. Additionally, we explored the importance of the H-bond donor effect from 7- and 8-OH groups of the coumarin ring by converting these groups to corresponding methoxy groups in the analog **25** (Scheme 2).

**Figure 3:**
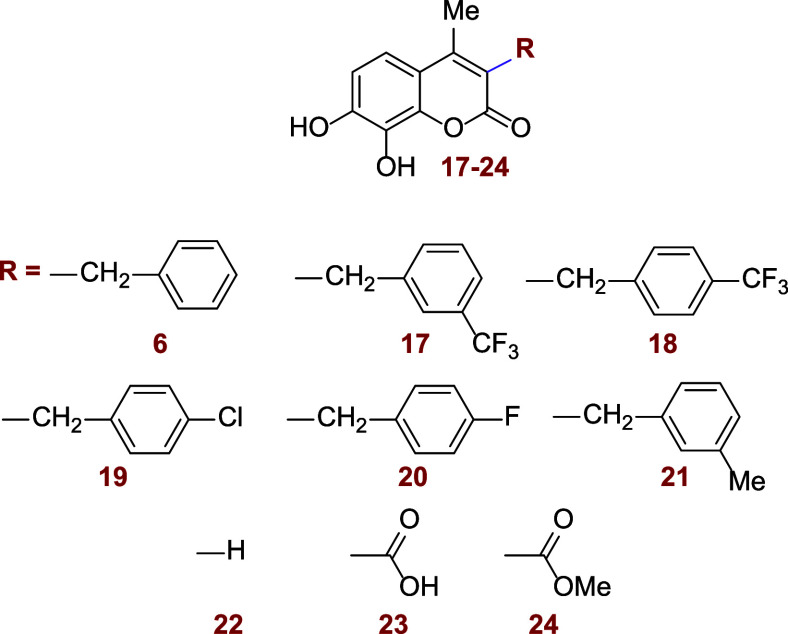
Library of eight coumarin analogs of compound **6**.

### Organic Synthesis

The syntheses of eight coumarins (**17–24**) is outlined in Schemes 1–3. We first developed a synthetic method to prepare compound **6** in 89% yield, which was then used to access five coumarin analogs (**17**-**21**) as shown in Scheme 1. First, ethyl acetoacetate (**26**) was alkylated with alkyl halides in the presence of NaH in THF to afford the alkylated compounds **27** in 71–86% yield. As these intermediate compounds were unstable, they were used directly in the next step without further purification. Reaction of alkylated products (**27**) with pyrogallol (**28**) in the presence of scandium triflate afforded five coumarins (**17**-**21**) in 40–62% yield.

**Scheme 1:**
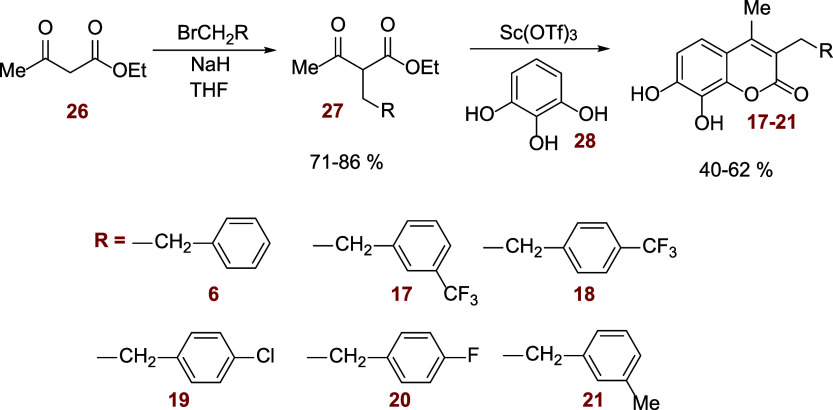
Synthesis of Coumarin Analogs **17**–**21**

The synthetic method used for the analogs **17**-**21** did not work for compounds **22**, **23**, and **24**, requiring alternate methods. Compounds **22** and **25** were synthesized starting from pyrogallol (**28**), as shown in Scheme 2. Pyrogallol was first acetylated using acetic anhydride in the presence of DMAP to afford pyrogallol triacetate (**29**) in 87% yield. Treatment of **29** with ethyl acetoacetate (**26**) in the presence of perchloric acid afforded compound **22** in 80% yield. Methylation of **22** with methyl iodide in the presence of K_2_CO_3_ in DMF furnished methylated analog **25** in 61% yield.

**Scheme 2:**
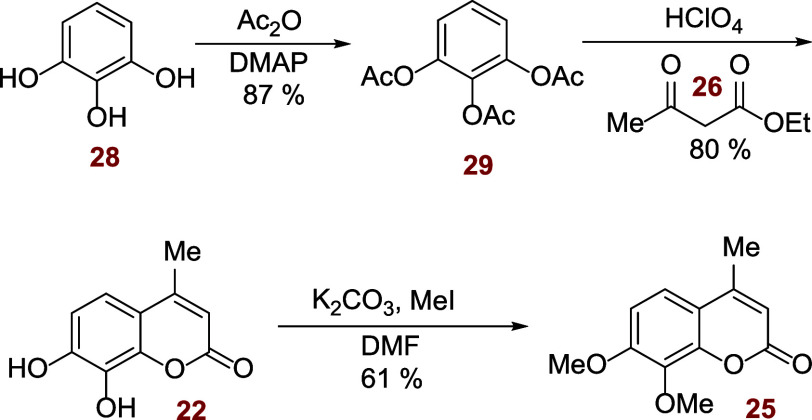
Synthesis of Coumarin Analogs **22** and **25**

Compounds **23** and **24** were synthesized from 2,3,4-trihydroxybenzaldehyde (**30**), as shown in Scheme 3. Treatment of **30** with Meldrum’s acid (**31**) in the presence of ammonium acetate in water gave the coumarin carboxylic acid derivative (**23**) in 93% yield. Fisher esterification of **23** with methanol in the presence of sulfuric acid afforded coumarin methyl ester analog **24** in 68% yield.

**Scheme 3:**
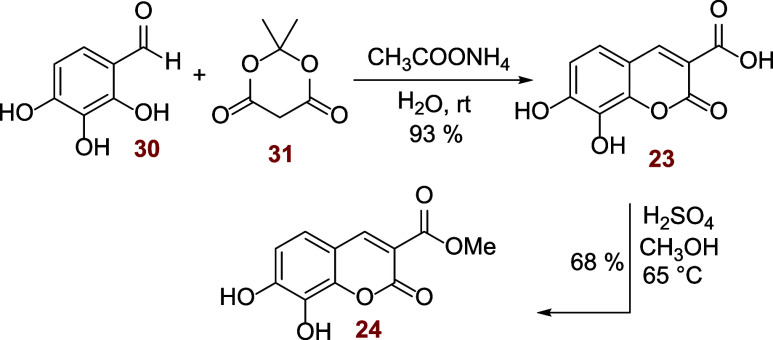
Synthesis of Coumarin Analogs **23** and **24**

All eight synthesized coumarins were first screened for *Sm*DAC inhibitory activity at 100 μM and the more active analogs were subjected to dose-dependent analysis and IC_50_ determination (Table 2). Compounds **20**, **21**, and **22** showed excellent *Sm*DAC inhibition at 100 μM with 79.5%, 80.7%, and 88.0%, respectively. Compounds **17**, **18**, and **19** showed moderate activity with 49.4%, 48.2%, and 67.5%, respectively. Others were weak inhibitors. In general, modifications to the *para* position of the benzene ring by introducing F (**20**) to Cl (**19**) to CF_3_ (**18**) groups gradually decreased the activity to 79.5% to 67.5% to 48.2%, respectively. Making the *meta* position more lipophilic by introducing a methyl group (**21**) slightly decreased the activity to 80.7% while introducing CF_3_ (**17**) resulted in significant reduction in activity to 49.4%. Lastly, methylation of 7-OH and 8-OH groups of the coumarin ring drastically reduced the enzymatic inhibitory activity from 88% (**22**) to 1.2% (**25**), further confirming the importance of 7-OH and 8-OH groups for the activity. The three most active compounds **20**, **21**, and **22** were then subjected to dose-dependent studies and their IC_50_ values for *Sm*DAC inhibition were determined to be 7.50 μM, 8.03 μM, and 11.51 μM, respectively. Though the *Sm*DAC inhibition activity of these three compounds was slightly lower than the compound **6**, two of the analogs **20** and **21** displayed better lipophilicity as indicated by their CLogP values of 3.17 and 3.34, which is an important determinant for their antibacterial activity. The CLogP value for **22** was found to be lower than **6** at 1.42, suggesting that it is more hydrophilic. This observation is consistent with the higher solubility of **22** at 205.9 μg/mL compared to relatively low solubilities of **6**, **20**, and **21** at 15.7 μg/mL, 17.4 μg/mL, and 35.2 μg/mL, respectively.

**Table 2:**
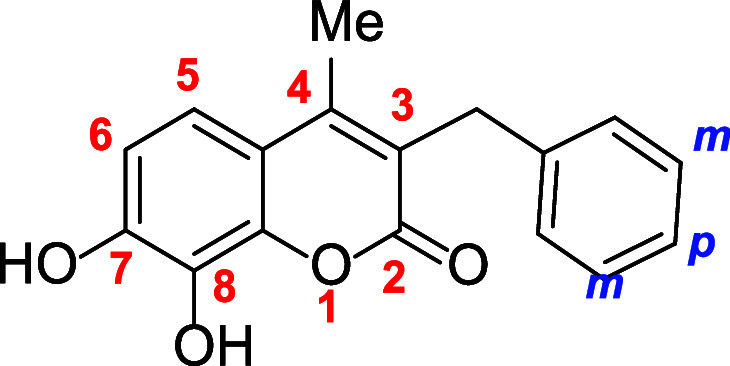

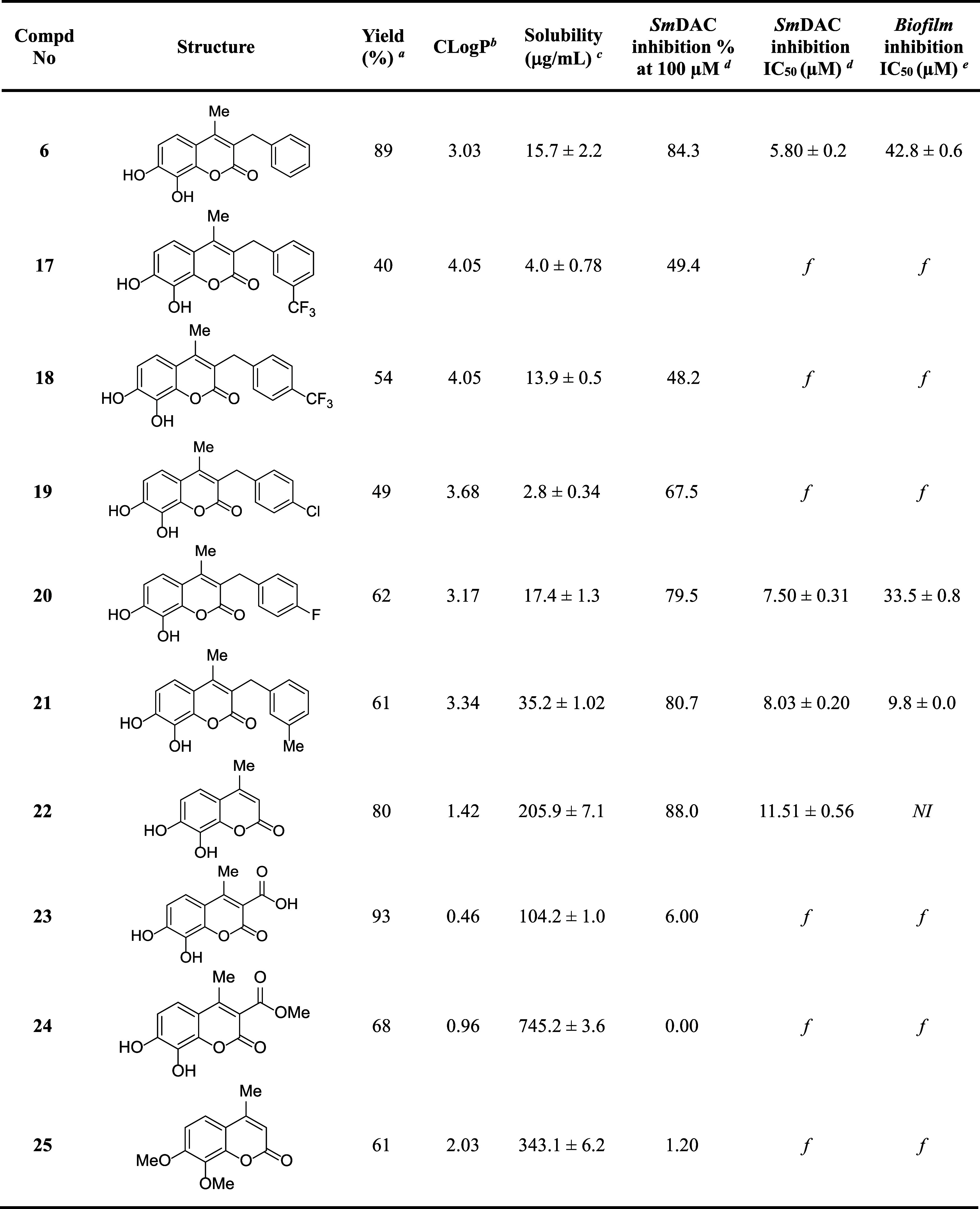
Results of Enzymatic Assay, Biofilm Assay, and Drug Properties of Synthesized Coumarins^a^ ^b^ ^c^ ^d^ ^e^ ^f^

a Yield of the purified compounds characterized by ^1^H NMR, ^13^C NMR, and HRMS.

b Calculated using Optibrium in SeeSAR 13.0.1.^45^

c Determined using a reported UV procedure^11^

d Determined by the HPLC enzymatic assay quantifying the ATP to c-di-AMP conversion by SmDAC.^24^

e Determined using a reported single-species *S. mutans* biofilm assay.^17,48^

f IC_50_ values were not determined for weak inhibitors, NI. No inhibition.

### SeeSAR Docking and HYDE Scoring

Active site interactions of the three most active analogs **6**, **20**, and **21**, and the initial compound STL372167 are shown in Figure 4A-D. Overall, all four coumarins displayed excellent binding affinity in the *Sm*DAC active site as shown by the larger number of favorable green coronas compared to unfavorable red coronas. The coumarin ring of all four analogs shared a set of favorable active site interactions. The 7-OH group on the coumarin ring showed two key H-bonds with the backbone amide CO (1.85 Å) group of Leu198 and the backbone amide NH (1.86 Å) group between Tyr197 and Leu198 residues. The lactone CO group of the coumarin ring H-bonded with the backbone amide NH (2.02 Å) group between Asp181 and Gly182 residues.

**Figure 4:**
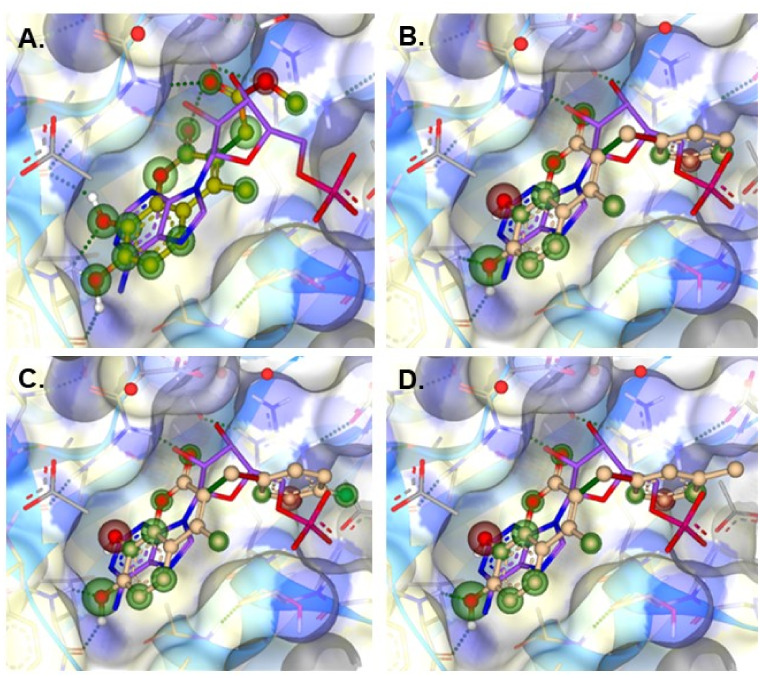
Docking models of STL372167 **(A)**, compound **6 (B)**, compound **20 (C)**, and compound **21 (D)** shown as wheat sticks in the in the AMP (purple sticks) binding site generated using *Sm*DAC crystal structure in SeeSAR 13.0.1. HYDE scoring-derived halos indicate favorable (green corona) and unfavorable (red corona) binding interactions.

Unlike STL372167, analogs **6**, **20**, and **21** displayed a few more unfavorable interactions within the active site due to the bulkiness of the benzyl group, which forces the molecule to take a slightly different orientation in the active site resulting in steric clashes. For example, the O atom of 8-OH in analog **6** showed unfavorable interaction as displayed by the large red corona 3.21 Å away from the CO of the amide NH connecting Ala196 and Tyr197. Similarly, the 8-OH group in analog **20** showed a large red corona that is 3.20 Å away from the CO group of the amide connecting Ala196 and Tyr197 and the *meta* carbon of the benzyl group displayed a small red corona due to the unfavorable interaction with the amide backbone NH connecting Thr212 and Arg213. In the same vein, in the methyl analog **21**, the 8-OH group showed an unfavorable interaction as demonstrated by the large red corona that is 3.21 Å away from the CO group of the amide NH group connecting Ala196 and Tyr197, and a small red corona on the *meta* carbon of the benzyl group due to an unfavorable interaction with the NH connecting Thr212 and Arg213. However, these unfavorable interactions were relatively weak compared to the strong binding interactions arising from the coumarin ring and by the *ortho* and *para* positions of the side chain benzene ring.

Compounds **20** and **21** displayed better biofilm inhibition than **6** with IC_50_ values of 33.5 μM and 9.8 μM, respectively (Table 2, Figure 5A). The biofilm inhibitory activities of the most active compound **21** were further investigated by fluorescence microscopy imaging (Figure 5C). SYTO9 staining showed significant reduction of biofilms at 5 μM and almost complete disappearance at 10 μM (Figure 5C, panel I). In addition, the presence of glucans was also significantly reduced at 5 μM and completely disappeared at 10 μM (Figure 5C, panel II). Furthermore, eDNA in *S. mutans* biofilms also showed noticeable reduction at 5 μM and a complete reduction at 10 μM (Figure 5C, panel III).

**Figure 5:**
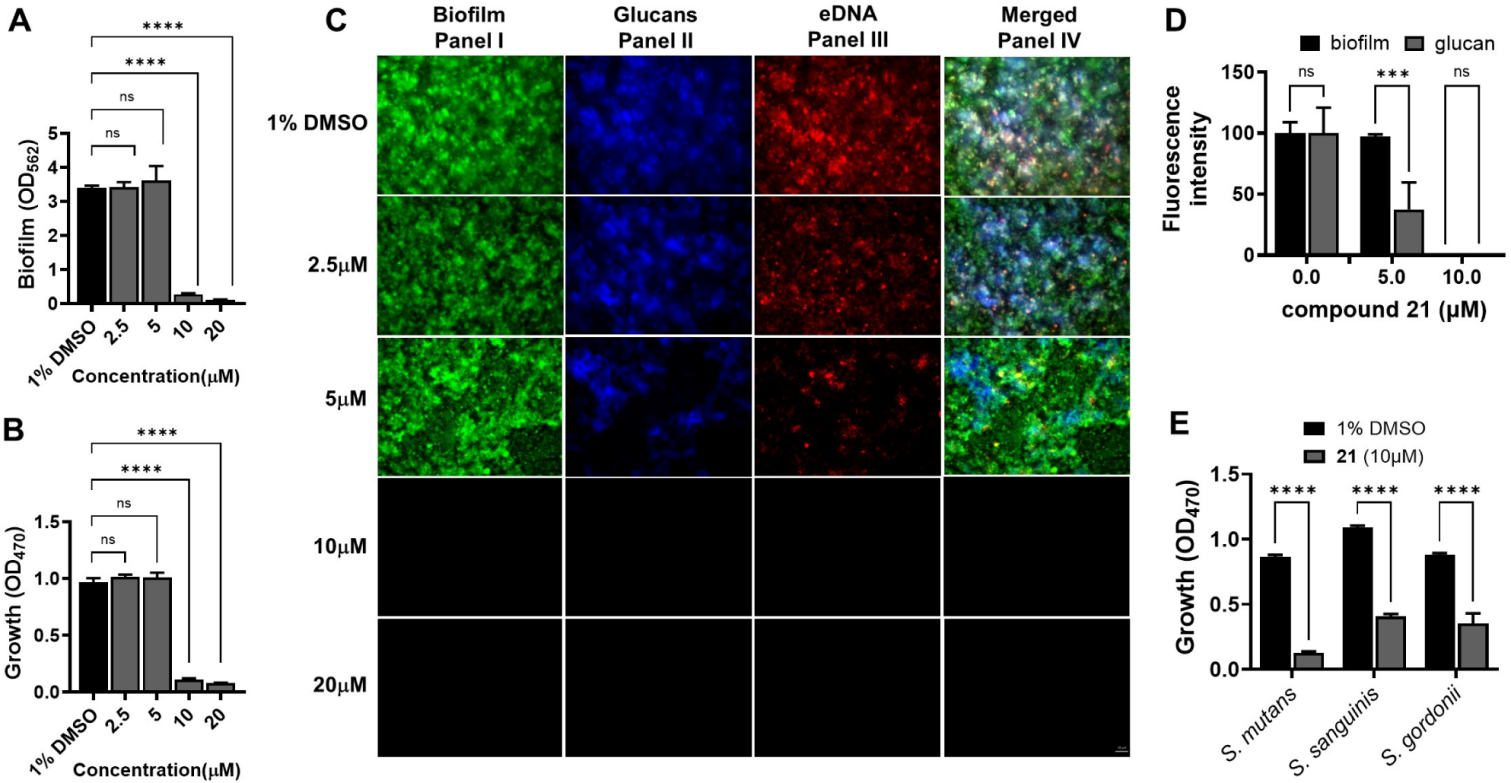
**A)** *S. mutans* UA159 treated with compound **21** at indicated concentrations and biofilm biomass quantified at OD_562_ using the crystal violet assay. **B)** *S. mutans* UA159 treated with compound **21** at indicated concentrations and the planktonic growth quantified at OD_470_. **C)** Representative fluorescence micrographs of UA159 biofilms after 16 h of treatment with compound **21**. Bacterial cells stained using SYTO9 (green, panel I); glucans stained using Cascade Blue–dextran conjugated dye (blue, panel II); eDNA stained using propidium iodide (red, panel III) and merged micrograph (panel IV). Scale-bar is 50 μM. **D)** The bacteria-to-glucan ratio calculated from the microscopy images of biofilm and glucans after treatment with compound **21**. **E)** *S. mutans* UA159, *S. gordonii* DL1, or *S. sanguinis* SK36 were treated with compound **21** at 10 μM, and the planktonic growth was measured at OD_470_. Data for biofilm and growth from three independent experiments. Statistical significance was tested with one-way ANOVA. ns = *p* ≥ 0.05, *** = *p* < 0.001, and **** = *p* < 0.0001.

The bacteria-to-glucan ratio from the microscopy images of the most active compound **21** showed relatively lower glucan levels compared to the amount of bacteria in the biofilm (Figure 5D), suggesting that the compound inhibits the glucan production rather than other aspects of the biofilm formation such as bacterial adhesion. Compound **21** did inhibit the growth of *S. mutans* similar to its inhibition of biofilm (Figure 5B), indicating lack of selectivity toward biofilm inhibition. However, compound **21** was found to be less bactericidal to the commensal streptococci *S. gordonii* and *S. sanguinis* inhibiting their growth by 60–70% at the treatment dose of 10 μM (Figure 5E). These effects were relatively less compared to about 90% inhibition observed for *S. mutans* at this dose, suggesting that compound has some selectivity toward preserving commensal species versus pathogenic *S. mutans*.

Interestingly, despite being a potent inhibitor of *Sm*DAC activity with an IC_50_ value of 11.51 μM, compound **22** failed to inhibit the growth or biofilm of *S. mutans* at 100 μM. The lack of antimicrobial activity of **22** could be attributed to the low lipophilicity and the associated poor cell membrane permeability of this molecule as indicated by its low CLogP of 1.42.

### Exogenous *Sm*DAC Rescues *S. Mutans* UA159 Growth and Biofilm Formation

Additional studies have been carried out to establish a link between *Sm*DAC inhibition and the observed biofilm phenotype by adding exogenous recombinant *Sm*DAC into the assay wells after the treatment with compound **21**. We observed that the addition of 1 μM *Sm*DAC after the compound treatment rescued about 50% of the ability of *S. mutans* to form biofilms and the addition of 2 μM *Sm*DAC rescued that ability by nearly 100% (Figure 6A, panel I). A similar but more pronounced rescuing effect on the ability of *S. mutans* to produce glucans was also observed . While the addition of 1 μM of *Sm*DAC did not rescue the glucan production, addition of 2 μM of *Sm*DAC fully rescued the bacteria’s ability to form glucans (Figure 6A, panel II). Similar rescue effects in eDNA levels of *S. mutans* were also observed, even though the effect was slightly less pronounced with the addition of 2 μM of *Sm*DAC (Figure 6A, panel III). These results were further corroborated by crystal violet quantification of biofilm biomass at OD_562_ and planktonic growth measurements at OD_470_. Addition of 1 μM *Sm*DAC after the compound treatment rescued about 70% of *S. mutans*’ ability to form biofilms and the addition of 2 μM *Sm*DAC rescued that ability by nearly 100% (Figure 6B). Addition of 1 μM *Sm*DAC after the compound treatment rescued planktonic growth by about 50% and the addition of 2 μM *Sm*DAC rescued that ability by about 80% (Figure 6C). These results further establish the direct link between the inhibition of *Sm*DAC and the antimicrobial activities of compound **21**.

**Figure 6:**
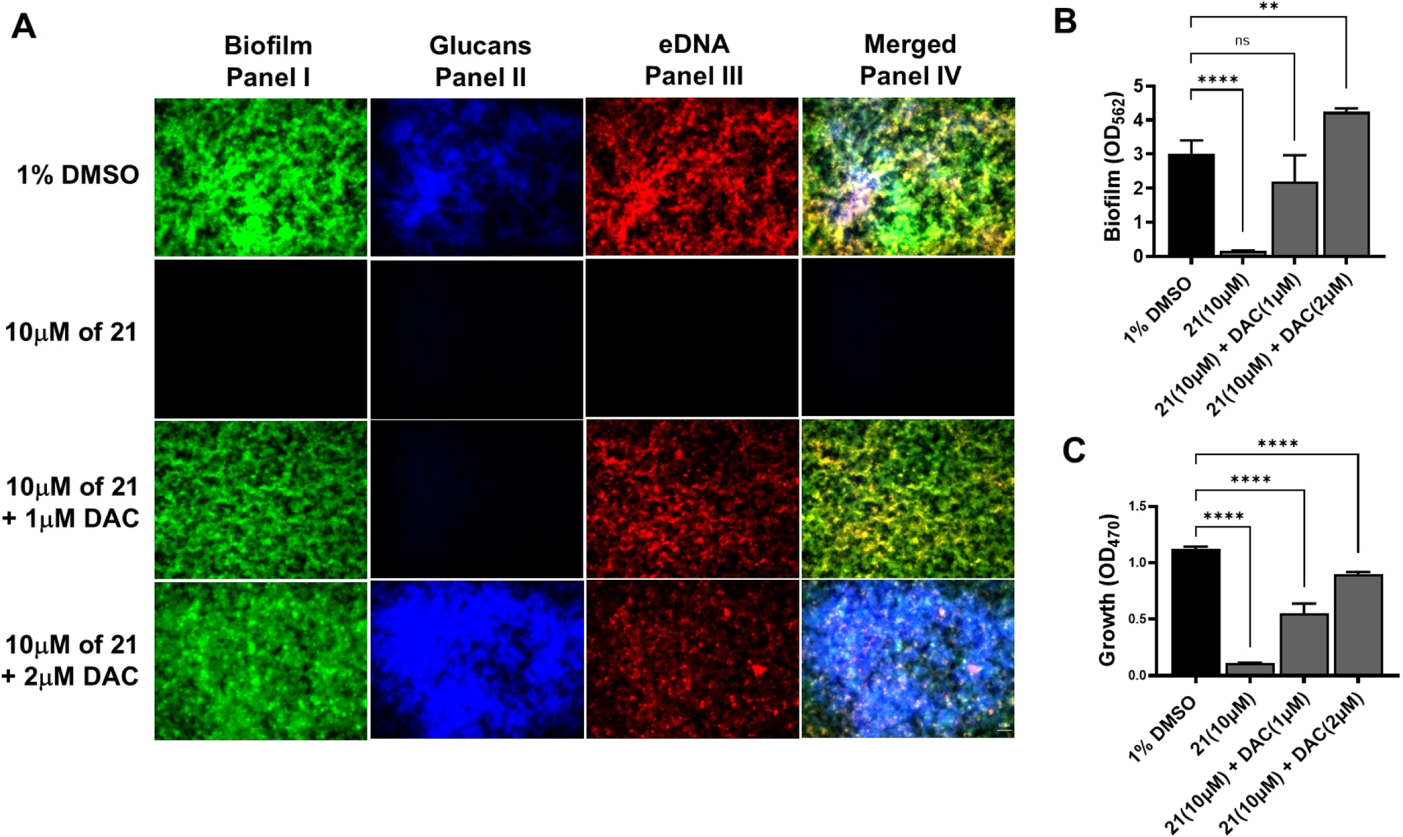
Exogenous *Sm*DAC rescues *S. mutans* UA159 growth and biofilm formation inhibited by compound **21**. **A)** Representative fluorescence micrographs of 16-h UA159 biofilms treated with Control (1% DMSO), 10 μM compound **21**, and 10 μM compound **21** supplemented with 1 μM or 2 μM *Sm*DAC. Panels show (I) SYTO9-stained bacterial cells (green), (II) Cascade Blue–dextran-labeled extracellular glucans (blue), (III) propidium iodide-stained eDNA (red), and (IV) merged images. **B)** Biofilm biomass quantification at OD_562_ using the crystal violet assay. **C)** Planktonic growth measured at OD_470_ and data represent the mean ± SD of three independent experiments. Statistical significance was tested with one-way ANOVA. ns = *p* ≥ 0.05, ** = *p* < 0.01, and **** = *p* < 0.0001.

### Mixed-Species Biofilm Inhibition

The selectivity of compound **21** for the inhibition of *S. mutans* biofilm and growth was further demonstrated in a mixed-species biofilm model established with *S. mutans*, *S. gordonii*, and *S. sanguinis*. *S. mutans* UA159 strain carrying a chromosomal mCherry label (UA159-mCherry) was used in these studies for selectively observing the effects on *S. mutans* in the mixed-species biofilm. Compound **21** at 10 μM significantly inhibited the growth of UA159-mCherry in single-species culture, whereas about 15% inhibition of growth was observed in the mixed-species community (Figure 7A), suggesting that compound **21** inhibited the growth of *S. mutans* more than the growth of representative commensals when tested at this concentration. In mixed-species biofilms measured by fluorescence, the overall matrix showed no significant difference between the control and treatment in mixed-species biofilms when treated with 10 μM of **21**, while it markedly reduced the level of biofilms produced by UA159-mCherry (Figure 7B-D). These results suggest that the biofilm inhibitory effects of compound **21** are more selective towards *S. mutans.*

**Figure 7:**
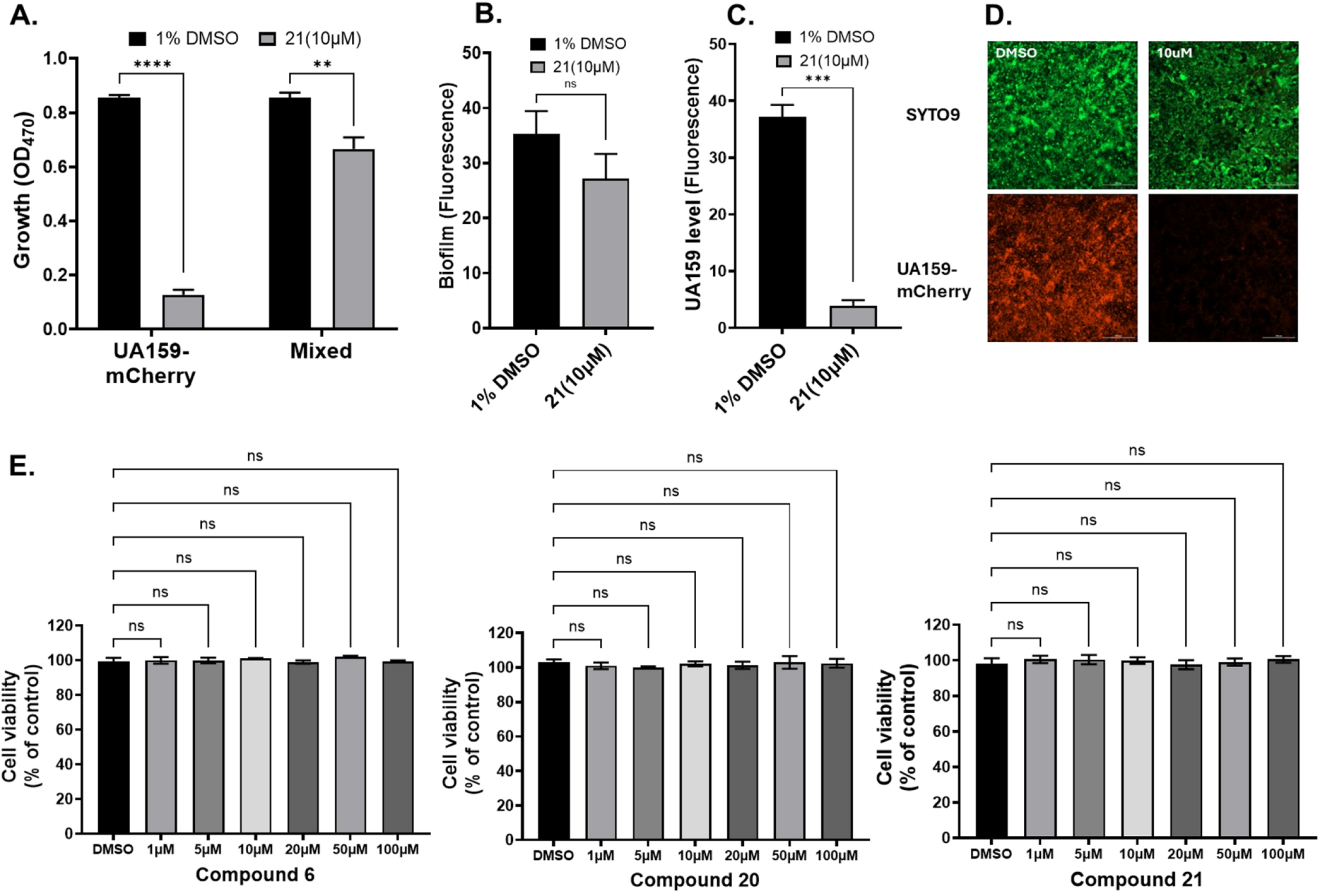
**A)** Treatment with 10 μM of compound **21** significantly inhibited the growth of *S. mutans* UA159 in single-species culture, whereas about 15% inhibition of growth was observed in the mixed-species community of *S. mutans*, *S. gordonii*, and *S. sanguinis*. **B)** In mixed-species biofilms, the biofilm biomass visualized using SYTO9 staining showed no significant difference between the control and treatment with 10 μM compound **21**. **C)** In mixed-species biofilms, treatment with 10 μM compound **21** markedly reduced the level of UA159-mCherry. **D)** Representative SYTO9 images of biofilm matrix and corresponding UA159-mCherry signals are shown for the control and compound treatment groups. Data for biofilm and growth from three independent experiments. **E)** Cell viability against oral gingival TIGK cells at various treatment concentrations of compounds **6**, **20**, and **21**. The cell viability data collected from three independent experiments. Statistical significance was tested with one-way ANOVA. ns = *p* ≥ 0.05, * = *p* < 0.05, ** = *p* < 0.01, *** = *p* < 0.001, and **** = *p* < 0.0001.

### Cytotoxicity Evaluation against TIGK Cells

Toxicity is not a major concern for our biofilm inhibitors as they are being developed as topical treatments for dental caries. However, considering that these compounds may have potential use in remediating oral biofilms in a clinical setting where there is a chance for accidental ingestion, it is important to demonstrate their safety and therapeutic utility early in the studies. As a preliminary evaluation of toxicity, we screened three of our lead compounds (**6**, **20**, and **21**) for cytotoxicity against normal oral gingival TIGK cells at a range of concentrations from 0 to 100 μM. None of the tested compounds were found to be cytotoxic to TIGK cells up to the highest treatment concentration of 100 μM (Figure 7E), suggesting that the compounds are nontoxic up to the tested dose.

## Conclusions

In conclusion, using structure-based drug design followed by similarity search and SAR explorations, we identified coumarins as *Sm*DAC inhibitors and advanced this chemotype to improved enzymatic inhibitory potency. Lead coumarins inhibited *S. mutans* biofilm formation and planktonic growth at low micromolar concentrations. Notably, biofilm inhibition marginally exceeded growth inhibition at tested doses, indicating relative biofilm selectivity and supporting the strategy of targeting c-di-AMP production to disrupt formation of cariogenic biofilms. The optimized compound **21** inhibited growth of representative commensal streptococci to a lesser degree compared to *S. mutans*. Additionally, inhibitor **21** minimally affected the growth and did not inhibit the biofilm of representative commensal streptococci in mixed-species assays at 10 μM, suggesting that it has potential to preserve commensal species in the oral microbiome. Furthermore, three lead compounds were not cytotoxic to TIGK cells up to the treatment concentration of 100 μM, suggesting that they are nontoxic and support their potential therapeutic applicability. While additional work is needed to fully define the mechanism of biofilm inhibition by coumarins, our data are consistent with the previously reported hypothesis of *Sm*DAC inhibition lowering intracellular c-di-AMP, thereby attenuating gtf expression and glucan production processes that are central to *S. mutans* biofilm development.^22^

## Experimental Section

### General Considerations

Bruker Avance Neo 400 and Bruker Avance Neo 300 NMR machines were used to record ^1^H NMR and ^13^C NMR spectra. Calibration of NMR peaks was carried out using TMS or appropriate solvent signals as internal standard. The chemical shift values are presented in ppm, and the coupling constants (*J*) are given in hertz (Hz). HRMS were recorded using Waters AutoSpec-Ultima NT magnetic sector mass spectrometer with Electron Impact (EI) ionization source. Anhydrous solvents were purchased in Sure-Seal bottles from Sigma-Aldrich chemical company. Other chemical reagents were purchased from Sigma-Aldrich or Fisher chemical companies and used as received. Silica gel plates with fluorescent indicators (Silicycle, UV254, 25 μm plates) were used to monitor the progress of chemical reactions. The crude products of the reactions were purified by column chromatography using Si gel (32–63 μm) from Dynamic Absorbent, Inc. Melting points were determined on a Mel-Temp II melting point apparatus and are uncorrected. All tested compounds have ≥95% purity as determined by HPLC. ATP (Thermo Scientific, Cat # R0441) and c-di-AMP (InvivoGen, Cat # tlrl-nacda) were purchased commercially, and aliquots were stored at −20 °C. Aliquots of 112 mg/mL *S. mutans* diadenylate cyclase (*Sm*DAC) in 20 mM Tris (pH 8.0), 100 mM NaCl were stored at −80 °C. Diluted stock solutions of *Sm*DAC were freshly prepared using the calculated MW = 17,750.24 Da.

### Methods of Biological Studies

#### Expression System and Purification of *Sm*DAC

The DNA fragment encoding *Sm*DAC (residues 116–265) was amplified by PCR from *S. mutans* genomic DNA and cloned into the pET21a expression vector (Novagen) to produce a C-terminally His-tagged recombinant construct. The construct was then expressed in *E. coli* BL21­(DE3). Cultures were grown in LB medium at 37 °C until an OD_600_ of 0.6 was reached. At this point, the protein expression was induced by the addition of 0.1 mM isopropyl β-D-1-thiogalactopyranoside (IPTG). Following induction, the cells were incubated at 18 °C for 16 h and subsequently harvested by centrifugation. The recombinant *Sm*DAC protein was first purified from cell lysates using nickel-nitrilotriacetic acid (Ni-NTA) affinity column (Cytiva) through AKTA prime purification system (GE). For further purification, the protein was subjected to size-exclusion chromatography using GF200 column equilibrated with buffer consisting of 20 mM Tris-HCl (pH 8.0) and 100 mM NaCl. The purity of the final protein was confirmed by Coomassie blue-stained SDS-PAGE. Purified *Sm*DAC was concentrated to 30 mg/mL, and this solution was stored at −80 °C.

#### HPLC Enzymatic Assay to Monitor c-di-AMP Production by*Sm*DAC^24^

Enzymatic reactions with a total volume of 400 μL, which contained 100 μM of ATP, 5 μM of *Sm*DAC, and 10% DMSO in the buffer (40 mM HEPES, pH 7.5, 100 mM NaCl, 10 mM MnCl_2_) were incubated on a heating block statically at 30 °C for 4 h. The reactions were then inactivated by heat-shocking at 95 °C for 10 min and allowed to stay at room temperature for 15 min. It was then transferred to 3 kDa centrifuge spin filters (VWR, Cat# 82031–344), centrifuged at 10,000 rpm for 30 min, and the filtrate (300 μL) was transferred to HPLC vials. Fifteen μL of this filtrate was injected into HPLC to detect ATP and c-di-AMP peaks. A Kinetex 5 μm C18 100 Å, LC Column 150 × 4.6 mm (Phenomenex, Cat# 00F-4601-E0) was used in the HPLC. The percentage of c-di-AMP produced in each reaction was determined based on the ratio of the peak area of c-di-AMP to the peak area of ATP. The peaks of ATP and c-di-AMP were compared to commercial standards for confirmation.

#### HPLC Method Used in the Enzymatic Assay

0–22 min (1% to 13% CH_3_CN/100 mM TEAA), 22–24.5 min (13% to 90% CH_3_CN/100 mM TEAA); 24.5–28.5 min (90% CH_3_CN/100 mM TEAA); 28.5–28.51 min (90% to 1% CH_3_CN/100 mM TEAA); 28.51–36 min (1% CH_3_CN/100 mM TEAA). Flow rate: 0.75 mL/min. Oven temperature: 30 °C. HPLC signals were detected at 254 nm UV.

#### HPLC Method for Compound Purity Analysis

HPLC analysis of the final compounds was conducted using Kinetex 5 μm C18 100 Å, LC Column 150 × 4.6 mm, compound = 3 mM, 10 μL injection volume, solvent: mobile phase buffer, Conditions: An isocratic solvent system of 60% CH_3_CN/40% H_2_O was used for a run time of 0–10 min. HPLC signals were detected at 254 nm UV. A chromatogram of mobile phase buffer (20 μL) was obtained for comparison.

### Bacterial Strains and Culture Conditions

*Streptococcus* strains used in this study are *S. mutans* UA159, *S. gordonii* DL1, and *S. sanguinis* SK36. They were grown statically for 24 h at 37 °C under 5% CO_2_ in freshly autoclaved Todd Hewitt Broth (THB) media (5 mL).

### Single-Species Biofilm Inhibition Assay^17,48^

*S. mutans* UA159 were grown independently for 24 h at 37 °C under 5% CO_2_ atmosphere in freshly autoclaved THB (5 mL). This overnight culture was diluted 1:1000 into chemically defined medium, CDM (1 mL) containing 1% sucrose and 1% DMSO. Then, the inhibitors at serially diluted concentrations in CDM were combined with the culture and transferred to 96-well plates. After incubating for 16 h at 37 °C, under 5% CO_2_ atmosphere, the planktonic growth of cells was quantified at OD_470_, and the biofilms were gently rinsed with H_2_O three times, dried, and then stained with 0.1% crystal violet (200 μL) for 10 min. The plates were then gently rinsed with H_2_O three times, dried, treated with 30% HOAc (200 μL), and placed on an orbital shaker (430 rpm) at room temperature for 30 min. The biofilm biomass was then quantified at OD_562_.

### Fluorescence Microscopy of *S. mutans* Biofilms

*S. mutans* UA159 was cultured for 24 h at 37 °C under 5% CO_2_ atmosphere in freshly autoclaved THB (5 mL). The overnight culture was then diluted 1:1000 into CDM (1 mL) containing 1% sucrose, 1% DMSO, and 1 μM dextran Cascade Blue (10,000 MW, anionic, lysine flexible) in 96-well plates. After incubation for 16 h at 37 °C under 5% CO_2_ atmosphere the biofilms were gently washed with H_2_O three times, dried, and wells were stained with 1X PBS (200 μL) containing 1 μM SYTO9 green-fluorescent nucleic acid stain and 2 μg/mL propidium iodide, covered in aluminum foil, and placed on an orbital shaker (430 rpm) at room temperature. After shaking for 10 min, the stained biofilms were visualized with a 4X objective using a Nikon Eclipse Ts2 integrated with NIS-Elements BR 5.41.01 imaging software. GFP green light, RFP red light, and DAPI blue light were used to visualize bacterial cells, eDNA, and glucans, respectively. Images are representative of three independent experiments performed on different days.

### Mixed-Species Biofilm and Growth Assay

Overnight cultures of *S. mutans* (mCherry strain), *S. gordonii* DL1, and *S. sanguinis* SK36 were subcultured in THB and grown to midexponential phase (OD_600_ = 0.6). For growth and biofilm assays, the cells were transferred to Chemically Defined Medium (CDM) supplemented with 1% sucrose and 1% DMSO. For the growth inhibition assay, cells were diluted 1:1000 in CDM as either single-species or multispecies inocula and treated with 10 μM of compound **21** in 96-well plates. After 16 h of incubation at 37 °C under 5% CO_2_ atmosphere, total growth was quantified by measuring absorbance at OD_600_.

To evaluate biofilm formation, the bacteria were diluted 1:1000 and cultured in the supplemented CDM and incubated at 37 °C under 5% CO_2_ atmosphere for 16 h. Then, the supernatant was removed, and the biofilms were gently washed with water three times to remove planktonic cells. The remaining biofilm biomass was stained with SYTO9 and imaged using Cytation 5 Cell Imaging Multi-Mode Reader. Fluorescence intensity was quantified using ImageJ software. All data are presented as mean ± S.D., and statistical significance was determined by an unpaired Student’s *t* test.

### Biofilm Rescue Experiments

*S. mutans* UA159 was cultured for 24 h at 37 °C under 5% CO_2_ in freshly autoclaved THB (5 mL). The overnight culture was then diluted 1:1000 into CDM (1 mL) containing 1% sucrose, 1% DMSO, and 1 μM dextran Cascade Blue (10,000 MW, anionic, lysine flexible). Then, the inhibitors at serially diluted concentrations in CDM were combined with the culture and transferred to 96-well plates. For biofilm rescue experiments, recombinant *Sm*DAC was added to the wells and incubated for 16 h at 37 °C under 5% CO_2_ atmosphere. Then, the biofilms formed were gently washed with H_2_O three times, dried, and biofilms were stained with 1 μM SYTO9 green-fluorescent nucleic acid and 2 μg/mL propidium iodide in 200 μL of 1×PBS buffer, covered in aluminum foil, and placed on an orbital shaker (430 rpm) at room temperature. After shaking for 10 min, the stained biofilms were visualized with a 20X objective using Keyence microscope. GFP green light, RFP red light, and DAPI blue light were used to visualize bacterial cells, eDNA, and glucans, respectively. Images are representative of three independent experiments performed on different days.

### Cytotoxicity Assay

Oral gingival TIGK cells were cultured in KGM-gold medium (Lonza Bioscience) and seeded into 96-well plates at a density of 1 × 10^5^ cells per well. After incubation at 37 °C under 5% CO_2_ atmosphere for 20 h, the cells were treated with varying concentrations of the compounds, **6**, **20**, or **21**. Following an additional 20 h incubation, the medium was replaced with fresh KGB-gold medium containing 0.5 mg/mL MTT and the cells were incubated for 5 h. Absorbance was measured at 580 nm, with a simultaneous reading at 630 nm used as the reference. Cell viability was calculated as (OD_580_ – OD_630_) normalized to the control group. Statistical analysis was performed using one-way ANOVA.

### Statistical Analysis

The analyses of the *in vitro* experimental data were performed by Student’s *t* test, one-way ANOVA, and Dunnett’s multiple comparisons test using GraphPad Prism 10.0.3.^49^ Error bars denote standard error of the mean (SEM). Differences were significant with a value of *p* ≤ 0.05. Experiments were repeated at least in triplicate and independently.

### Chemistry Methods

#### *In Silico* Screening to Identify Leads as Competitive Inhibitors of*Sm*DAC

Small-molecule libraries from chemical databases (ChemDiv, Enamine, ZINC, and NCI) were downloaded and combined using Mona (Center for Bioinformatics of the University of Hamburg), a free interactive tool which enables the combination, splitting, filtering, chemical analysis, and file conversion of small-molecule data sets. The 1.1 M compound library thus generated was filtered based on Lipinski’s Rule of 5, except for MW. The filtered library was then split randomly into smaller libraries of 45K compounds, which were then exported as SDF files. KNIME Analytics Platform 4.7.4, a free open-source tool for data integration and analysis, coupled with SeeSAR 13.0 (BioSolveIT), was used to generate workflows for generating 3D coordinates, docking, sorting, and predicting Hyde scores of the split small-molecule libraries with the target protein’s binding site. Using SeeSAR 13.0 GUI, the docked libraries were further filtered using built-in filters (Drug-likeness: HBD ≤ 5, HBA ≤ 10, MW ≤ 500, and CLogP ≤ 5; Lead-likeness: HBD < 4, HBA < 7, rotatable bonds <8, rings <5, (HBD+HBA) < 10, Heavy atoms 10–27, and CLogP 0–4; and Estimated Binding Affinity <10,000 nM). The resulting small molecules from each library were then exported as SDF files, opened in Mona, and combined into a single library. This library was then redocked using KNIME Analytics Platform 4.7.4 and refiltered using SeeSAR 13.0s GUI built-in filters mentioned above, but with estimated binding affinity <3,000 nM. In SeeSAR 13.0 GUI, the results were further analyzed for their torsional strain, and intra- and intermolecular clashing. 66 small molecules with best predicted drug properties were purchased for the *in vitro* evaluation.

### Solubility Determination

The solubility of *Sm*DAC inhibitors was determined using a UV spectroscopy method reported in the literature.^11,50,51^ Initially, a stock solution of each compound was prepared in 1% DMSO, and its UV spectrum was recorded from 200 to 800 nm using a Cary 300 UV–vis spectrophotometer to determine λ_max_. Calibration curves were then constructed by using a series of known concentrations of the compound spanning the expected solubility range and measuring their absorbance at the λ_max_ in triplicate to ensure accuracy and reproducibility. The linearity of the calibration curve was confirmed, and the equation was used for subsequent calculations. For solubility determination, an excess of the compound was added to saturate the solution and allowed to equilibrate at 25 °C for 24 h with constant agitation. The resulting solutions were filtered through VWR centrifugal filter, and the filtrate was appropriately diluted so that its absorbance fell within the calibration range. The absorbance of these diluted samples was measured at λ_max_, and the concentration was back-calculated using the calibration curve and corrected for the dilution factor. The experiment was repeated in triplicate on different days.

### Synthesis

#### General Procedure A for Benzylation (**27**)

To a solution of ethyl acetoacetate (1 equiv) in THF (10 mL), NaH (1 equiv) was added and the reaction mixture was stirred under N_2_ atmosphere at room temperature for 30 min. Then benzyl bromide (1 equiv) was added slowly to the reaction mixture and stirring continued for 12 h. Upon completion of the reaction as determined by TLC (10% EtOAc in hexanes), the mixture was quenched with saturated solution of Na_2_SO_4_ and extracted using EtOAc (3 × 20 mL). The combined organic extract was washed with water (2 × 30 mL), brine (1 × 30 mL), and dried over anhydrous Na_2_SO_4_. The drying agent was filtered off, and the filtrate was concentrated under reduced pressure to afford the products **27** in 71–86% yield. Due to the instability of the products, they were used directly for the next step without further purification.

#### General Procedure B for Coumarin Analogs (**6** and **17**–**21**)

Pyrogallol (1 equiv) and Sc­(OTf)_3_ (0.1 equiv) were added to **27** (1.2 equiv). The neat reaction mixture under N_2_ atmosphere was heated at 85 °C for 30 min. Upon the completion of the reaction as shown by TLC (50% EtOAc in hexanes), the mixture was diluted using EtOAc (60 mL) and washed with water (3 × 30 mL), brine (1 × 30 mL), and dried over Na_2_SO_4_. The drying agent was filtered off, and the filtrate was concentrated under reduced pressure to afford the crude products, which were purified by column chromatography over Si gel using 20–50% EtOAc in hexanes to afford the pure products **6** and **17**–**21** in 40–89% yield.

#### 3-Benzyl-7,8-dihydroxy-4-methyl-2*H*-chromen-2-one (**6**)

(99 mg, 89%) as a white solid; Mp: 184–186 °C; ^1^H NMR (400 MHz, DMSO-d_6_): δ 2.37 (s, 3H), 3.93 (s, 2H), 6.80 (d, *J* = 8.8 Hz, 2H), 7.11–7.19 (m, 5H), and 7.21–7.28 (m, 2H); ^13^C NMR (100 MHz, DMSO-d_6_): δ 15.2, 32.1, 112.2, 113.1, 115.5, 119.9, 125.9, 127.9, 128.3, 131.9, 139.5, 142.1, 148.6, 148.8, and 161.2 ppm; HRMS calculated for C_17_H_14_O_4_ [M + H]^+^: 283.0970, found 283.0974.

#### 7,8-Dihydroxy-4-methyl-3-(3-trifluoromethylbenzyl)-2*H*-chromen-2-one (**17**)

(221 mg, 40%) as a white solid; Mp: 202–204 °C; ^1^H NMR (400 MHz, DMSO-d_6_): δ 2.39 (s, 3H), 4.03 (s, 2H), 6.81 (d, *J* = 8.8 Hz, 1H), 7.14 (d, *J =* 8.8 Hz, 1H), and 7.49–7.58 (m, 4H); ^13^C NMR (100 MHz, DMSO-d_6_): δ 15.2, 31.9, 112.2, 113.0, 115.7, 119.1, 122.8 − **C** –C–CF_3_ (q, *J* = 4 Hz), 124.5 −**C**–C–CF_3_ (q, *J* = 4 Hz), 129.0 −C– **C** –CF_3_ (q, *J* = 31 Hz), 124.2 −C–C– **C** F_3_ (q, *J* = 270 Hz), 131.9, 132.0, 141.1, 142.2, 148.8, 159.5, and 161.2 ppm; HRMS calculated for C_18_H_14_O_4_F_3_ [M + H]^+^: 351.0844, found 351.0840.

#### 7,8-Dihydroxy-4-methyl-3-(4-trifluoromethylbenzyl)-2*H*-chromen-2-one (**18**)

(149 mg, 54%) as a white solid; Mp: 218–221 °C; ^1^H NMR (400 MHz, DMSO-d_6_): δ 2.38 (s, 3H), 4.02 (s, 2H), 6.81 (d, *J* = 8.4 Hz, 1H), 7.14 (d, *J =* 8.8 Hz, 1H), 7.43 (d, *J* = 8 Hz), and 7.62 (d, *J* = 8 Hz, 2H); ^13^C NMR (100 MHz, DMSO-d_6_): δ 15.3, 32.1, 112.2, 113.1, 115.7, 119.0, 125.2 − **C** –C–CF_3_ (q, *J* = 4 Hz), 126.8 −C– **C** –CF_3_ (q, *J* = 31 Hz), 124.4 −C-C- **C** F_3_ (q, *J* = 270 Hz), 128.8, 131.9, 142.2, 144.5, 148.8, 149.6, and 161.2 ppm; HRMS calculated for C_18_H_14_O_4_F_3_ [M + H]^+^: 351.0844, found 351.0844

#### 3-(4-Chlorobenzyl)-7,8-dihydroxy-4-methyl-2*H*-chromen-2-one (**19**)

(244 mg, 49%) as a white solid; Mp: 209–212 °C; ^1^H NMR (400 MHz, DMSO-d_6_): δ 2.37 (s, 3H), 3.92 (s, 2H), 6.81 (d, *J =* 8.8 Hz, 1H), 7.13 (d, *J =* 8.8 Hz, 1H), 7.23 (d, *J =* 8.4 Hz, 1H), and 7.31 (d, *J =* 8.4 Hz, 1H); ^13^C NMR (100 MHz, DMSO-d_6_): δ 15.2, 31.5, 112.2, 113.1, 115.6, 119.5, 128.3, 129.8, 130.5, 131.9, 138.5, 142.1, 148.7, 149.1, and 161.2 ppm; HRMS calculated for C_17_H_14_O_4_Cl [M + H]^+^: 317.0581, found 317.0586

#### 3-(4-Fluorobenzyl)-7,8-dihydroxy-4-methyl-2*H*-chromen-2-one (**20**)

(149 mg, 62%) as a white solid; Mp: 183–185 °C; ^1^H NMR (400 MHz, DMSO-d_6_): δ 2.37 (s, 3H), 3.92 (s, 2H), 6.80 (d, *J =* 8.8 Hz, 1H), 7.05–7.14 (m, 3H), 7.22–7.26 (m, 2H), 9.25 (bs, 1H, OH), and 9.98 (bs, 1H, OH); ^13^C NMR (100 MHz, DMSO-d_6_): δ 15.2, 31.3, 112.2, 113.1, −C–C– **C** –C–F 115.0 (d, *J* = 21 Hz), 115.6, 119.8, −C– **C** –C–C–F 129.7 (d, *J* = 8 Hz), 131.9, − **C** –C–C–C–F 135.6 (d, *J* = 3 Hz), 142.1, 148.7, 148.9, −C–C–C– **C** –F 160.8 (d, *J* = 240 Hz), and 161.2 ppm; HRMS calculated for C_17_H_14_O_4_F [M + H]^+^: 301.0876, found 301.0885

#### 7,8-Dihydroxy-4-methyl-3-(3-methylbenzyl)-2*H*-chromen-2-one (**21**)

(138 mg, 61%) as a white solid; Mp: 210–212 °C; ^1^H NMR (400 MHz, DMSO-d_6_): δ 2.23 (s, 3H), 2.35 (s, 3H), 3.89 (s, 2H), 6.80 (d, *J =* 8.8 Hz, 1H), 6.97–7.00 (m, 3H), 7.11–7.15 (m, 2H), 9.27 (bs, 1H, OH), and 9.98 (bs, 1H, −OH); ^13^C NMR (100 MHz, DMSO-d_6_): δ 15.2, 21.0, 32.0, 112.2, 113.2, 115.5, 119.9, 125.0, 126.6, 128.3, 128.8, 131.9, 137.4, 139.4, 142.1, 148.6, 148.9, and 161.3 ppm; HRMS calculated for C_18_H_17_O_4_ [M + H]^+^: 297.1127, found 297.1135

#### Benzene-1,2,3-triyl triacetate (**29**)

To a solution of pyrogallol (200 mg, 1.58 mmol) in Ac_2_O (1 mL) catalytic amount of DMAP (1.9 mg, 0.015 mmol) was added and stirred for 1 h under N_2_ atmosphere at room temperature. TLC examination using 10% MeOH in CHCl_3_ showed the completion of the reaction. Upon the completion of the reaction, the mixture was diluted using EtOAc (20 mL) and washed with water (3 × 20 mL), brine (1 × 20 mL), and dried over Na_2_SO_4._ The drying agent was filtered off and the filtrate was concentrated under reduced pressure to afford the pure product **29** as white solid (276 mg, 87%); Mp: 160–163 °C; ^1^H NMR (300 MHz, CDCl_3_): δ 2.30 (s, 6H), 2.31 (s, 3H), 7.12–7.15 (m, 2H), and 7.28–7.31 (m, 1H); ^13^C NMR (75 MHz, CDCl_3_): δ 20.2, 20.7, 120.7, 126.0. 134.7, 143.5, 167.0, and 167.9 ppm.

#### 7,8-Dihydroxy-4-methyl-2*H*-chromen-2-one (**22**)

Perchloric acid (5 mL) was added dropwise at room temperature to a mixture of **29** (200 mg, 5.07 mmol) and ethyl acetoacetate (205 mg, 10.1 mmol) and stirred for 6 h. TLC examination using 10% MeOH in CHCl_3_ showed the completion of the reaction. The reaction mixture was poured into a mixture of ice water (100 mL) while stirring. The resultant precipitate was filtered, and the collected solid was washed with water and dried, The crude compound thus obtained was recrystallized from MeOH to produce the desired compound **22** as a white solid (350 mg, 80%); Mp: 240–243 °C; ^1^H NMR (400 MHz, DMSO-d_6_): δ 2.35 (s, 3H), 6.12 (s, 1H), 6.80 (d, *J* = 8.8 Hz, 1H), and 7.09 (d, *J* = 8.8 Hz, 1H); ^13^C NMR (100 MHz, DMSO-d_6_): δ 18.2, 110.1, 112.0, 112.7, 115.4, 132.1,143.3, 149.3, 153.9, and 160.1 ppm; HRMS calculated for C_10_H_9_O_4_ [M + H]^+^: 193.0501, found 193.0501.

#### 7,8-Dimethoxy-4-methyl-2*H*-chromen-2-one (**25**)

To a solution of compound **22** (50 mg, 0.26 mmol) in DMF (2 mL), K_2_CO_3_ (143 mg, 1.03 mmol) and MeI (110 mg, 0.77 mmol) were added, and the reaction mixture was stirred for 12 h at room temperature under N_2_ atmosphere. Upon the completion of the reaction as shown by TLC (10% MeOH in CHCl_3_), the reaction mixture was diluted with EtOAc (30 mL) and washed with water (3 × 20 mL), brine (1 × 20 mL), and dried over Na_2_SO_4._ The drying agent was filtered off, and the filtrate was concentrated under reduced pressure to afford the pure product **25** (35 mg, 61%) as a white solid; Mp: 118–120 °C; ^1^H NMR (400 MHz, CDCl_3_): δ 2.39 (s, 3H), 3.95 (s, 3H), 3.98 (s, 3H), 6.15 (s, 1H), 6.88 (d, *J* = 8.8 Hz, 1H), and 7.29 (d, *J* = 8.8 Hz, 1H); ^13^C NMR (100 MHz, CDCl_3_): δ 18.9, 56.5, 61.6, 108.2, 112.5, 115.0, 119.6, 136.4, 147.9, 152.6, 155.5, and 160.7 ppm; Spectral data matched with the reported values.^52^

#### 7,8-Dihydroxy-2-oxo-2*H*-chromene-3-carboxylic acid (**23**)

A solution of 2,3,4-trihydroxybenzaldehyde **30** (1 g, 6.48 mmol), Meldrum’s acid **31** (1.40 g, 9.7 mmol), and NH_4_OAc in water (25 mL) was stirred for 4 h at room temperature. Upon the completion of the reaction as shown by TLC (50% EtOAc in hexanes), the reaction mixture was acidified with 1 N HCl to pH 2, and the precipitate formed was filtered off and recrystallized in MeOH to afford the pure product **23** (1.34 g, 93%) as a white solid; Mp: 282–284 °C; ^1^H NMR (400 MHz, DMSO-d_6_): δ 6.86 (d, *J* = 8.4 Hz, 1H), 7.26 (d, *J =* 8.4 Hz, 1H), 9.60 (bs, 1H, −OH) 10.68 (bs, 1H, −OH), and 12.80 (bs, 1H, −OH); ^13^C NMR (100 MHz, DMSO-d_6_): δ 111.3, 112.1, 113.3, 121.5, 131.8, 144.7, 149.9, 152.4, 157.7, and 164.2 ppm; HRMS calculated for C_10_H_6_O_6_ [M + H]^+^: 223.0243, found 223.0252.

#### Methyl 7,8-Dihydroxy-2-oxo-2*H*-chromene-3-carboxylate (**24**)

To a solution of **23** (200 mg, 1.11 mmol) in MeOH (8 mL), three drops of conc. H_2_SO_4_ were added, and the reaction mixture was refluxed at 70 °C for 12 h. Upon the completion of the reaction as shown by TLC (20% MeOH in CHCl_3_), the reaction mixture was diluted using EtOAc (30 mL) and washed with water (3 × 20 mL), brine (1 × 20 mL), and dried over Na_2_SO_4._ The drying agent was filtered off and the filtrate was concentrated under reduced pressure to afford the pure product **24** (142 mg, 68%) as a white solid; Mp: 205–207 °C; ^1^H NMR (400 MHz, DMSO-d_6_): δ 3.78 (s, 3H), 6.85 (d, *J =* 8.4 Hz, 1H), 7.25 (d, *J =* 8.4 Hz, 1H), and 8.64 (s, 1H); ^13^C NMR (100 MHz, DMSO-d_6_): δ 52.1, 111.2, 111.5, 113.3, 121.7, 131.8, 144.9, 150.3, 152.6, 156.4, and 163.6 ppm; HRMS calculated for C_11_H_9_O_6_ [M + H]^+^: 237.0399, found 237.0405.

## Supplementary Material



## Notes

Copies of ^1^H NMR spectra, ^13^C NMR spectra, and HPLC chromatograms (PDF)

## Abbreviations

AMP: adenosine monophosphate
ANOVA: analysis of variance
ATP: adenosine triphosphate
C-di-AMP: cyclic-diadenosine monophosphate
CDM: chemically defined medium
CLogP: calculated LogP
DAC: diadenylate cyclase
DMAP: *N*,*N*-dimethyl aminopyridine
DMF: *N*,*N*-dimethyl formamide
eDNA: extracellular DNA
EBE: electric-magnetic-electric
EI: electron Impact
Gtf: glucosyltransferase
HBA: hydrogen bond acceptor
HBD: hydrogen bond donor
HPLC: high-performance liquid chromatography
HRMS: high-resolution mass spectrum
IC_50_: half-maximal inhibitory concentration
MIC: minimum inhibitory concentration
NMR: nuclear magnetic resonance
SAR: structure–activity relationship;
*S. mutans*: *Streptococcus mutans*
*S. gordonii*: *Streptococcus gordonii*
*S. sanguinis*: *Streptococcus sanguinis*
THB: Todd-Hewitt Broth
THF: tetrahydrofuran
TLC: thin-layer chromatography
TMS: tetramethyl silane
UAB-EHS: The University of Alabama at Birmingham Environmental Health and Safety

## References

[1] Pitts N. B., Twetman S., Fisher J., Marsh P. D. (2021). Understanding dental
caries as a non-communicable disease. Br Dent
J..

[2] Chen X., Daliri E. B., Kim N., Kim J. R., Yoo D., Oh D. H. (2020). Microbial Etiology
and Prevention of Dental Caries: Exploiting Natural
Products to Inhibit Cariogenic Biofilms. Pathogens.

[3] Ito S., Misaki T., Naka S., Wato K., Nagasawa Y., Nomura R., Otsugu M., Matsumoto-Nakano M., Nakano K., Kumagai H. (2019). Specific strains of
Streptococcus mutans, a pathogen of dental caries, in the tonsils,
are associated with IgA nephropathy. Sci. Rep..

[4] Lemos J. A., Palmer S. R., Zeng L., Wen Z. T., Kajfasz J. K., Freires I. A., Abranches J., Brady L. J. (2019). The Biology of Streptococcus
mutans. Microbiol. Spectrum.

[5] Overman P. R. (2000). Biofilm:
A New View of Plaque. J. Contemp Dent Pract.

[6] Oyanagi T., Tagami J., Matin K. (2012). Potentials of Mouthwashes
in Disinfecting
Cariogenic Bacteria and Biofilms Leading to Inhibition of Caries. Open Dent J..

[7] Kolenbrander P. E. (2000). Oral Microbial
Communities: Biofilms, Interactions, and Genetic Systems. Annu. Rev. Microbiol..

[8] Ren Z., Chen L., Li J., Li Y. (2016). Inhibition of Streptococcus
mutans Polysaccharide Synthesis by Molecules Targeting Glycosyltransferase
Activity. J. Oral Microbiol..

[9] Ren Z., Cui T., Zeng J., Chen L., Zhang W., Xu X., Cheng L., Li M., Li J., Zhou X. (2016). Molecule Targeting Glucosyltransferase Inhibits Streptococcus mutans
Biofilm Formation and Virulence. Antimicrob.
Agents Chemother.

[10] Taubman M. A., Nash D. A. (2006). The Scientific and Public Health Imperative for a Vaccine
Against Dental Caries. Nat. Rev. Immunol..

[11] Ahirwar P., Kozlovskaya V., Nijampatnam B., Rojas E. M., Pukkanasut P., Inman D., Dolmat M., Law A. C., Schormann N., Deivanayagam C. (2023). Hydrogel-Encapsulated Biofilm Inhibitors Abrogate
the Cariogenic Activity of Streptococcus mutans. J. Med. Chem..

[12] Ahirwar P., Kozlovskaya V., Pukkanasut P., Nikishau P., Nealy S., Harber G., Michalek S. M., Antony L., Wu H., Kharlampieva E. (2024). Polymer vesicles for the delivery of inhibitors
of cariogenic biofilm. Dent Mater..

[13] Nijampatnam B., Ahirwar P., Pukkanasut P., Womack H., Casals L., Zhang H., Cai X., Michalek S. M., Wu H., Velu S. E. (2021). Discovery of Potent
Inhibitors of Streptococcus mutans
Biofilm with Antivirulence Activity. ACS Med.
Chem. Lett..

[14] Nijampatnam B., Casals L., Zheng R., Wu H., Velu S. E. (2016). Hydroxychalcone
Inhibitors of Streptococcus mutans Glucosyl transferases and Biofilms
as Potential Anticaries Agents. Bioorg. Med.
Chem. Lett..

[15] Scaffa P. M. C., Kendall A., Icimoto M. Y., Fugolin A. P. P., Logan M. G., De Vito-Moraes A. G., Lewis S. H., Zhang H., Wu H., Pfeifer C. S. (2023). The potential use of glycosyl-transferase inhibitors
for targeted reduction of S. mutans biofilms in dental materials. Sci. Rep..

[16] Zhang Q., Ma Q., Wang Y., Wu H., Zou J. (2021). Molecular Mechanisms
of Inhibiting Glucosyltransferases for Biofilm Formation in Streptococcus
mutans. Int. J. Oral Sci..

[17] Zhang Q., Nijampatnam B., Hua Z., Nguyen T., Zou J., Cai X., Michalek S. M., Velu S. E., Wu H. (2017). Structure-Based Discovery
of Small Molecule Inhibitors of Cariogenic Virulence. Sci. Rep..

[18] Veiga N., Figueiredo R., Correia P., Lopes P., Couto P., Fernandes G. V. O. (2023). Methods
of Primary Clinical Prevention of Dental Caries
in the Adult Patient: An Integrative Review. Healthcare.

[19] Commichau F. M., Heidemann J. L., Ficner R., Stülke J. (2019). Making and
Breaking of an Essential Poison: the Cyclases and Phosphodiesterases
That Produce and Degrade the Essential Second Messenger Cyclic di-AMP
in Bacteria. J. Bacteriol..

[20] Herzberg C., Meißner J., Warneke R., Stülke J. (2023). The many roles
of cyclic di-AMP to control the physiology of Bacillus subtilis. microLife.

[21] Mudgal S., Manikandan K., Mukherjee A., Krishnan A., Sinha K. M. (2021). Cyclic
di-AMP: Small molecule with big roles in bacteria. Microb. Pathog.

[22] Peng X., Zhang Y., Bai G., Zhou X., Wu H. (2016). Cyclic di-AMP
mediates biofilm formation. Mol. Microbiol..

[23] Galperin M. Y. (2023). All DACs
in a Row: Domain Architectures of Bacterial and Archaeal Diadenylate
Cyclases. J. Bacteriol..

[24] Rojas E. M., Zhang H., Velu S. E., Wu H. (2024). Tetracyclic
homoisoflavanoid
(+)-brazilin: a natural product inhibits c-di-AMP-producing enzyme
and Streptococcus mutans biofilms. Microbiol.
Spectrum.

[25] Stülke J., Krüger L. (2020). Cyclic di-AMP
Signaling in Bacteria. Annu. Rev. Microbiol..

[26] Flores-Morales V., Villasana-Ruíz A. P., Garza-Veloz I., González-Delgado S., Martinez-Fierro M. L. (2023). Therapeutic
Effects of Coumarins with Different Substitution Patterns. Molecules.

[27] Ibrar A., Shehzadi S. A., Saeed F., Khan I. (2018). Developing hybrid molecule
therapeutics for diverse enzyme inhibitory action: Active role of
coumarin-based structural leads in drug discovery. Bioorg. Med. Chem..

[28] Sharifi-Rad J., Cruz-Martins N., López-Jornet P., Lopez E. P., Harun N., Yeskaliyeva B., Beyatli A., Sytar O., Shaheen S., Sharopov F. (2021). Natural Coumarins: Exploring the Pharmacological
Complexity and Underlying Molecular Mechanisms. Oxid. Med. Cell. Longev..

[29] Stefanachi A., Leonetti F., Pisani L., Catto M., Carotti A. (2018). Coumarin:
A Natural, Privileged and Versatile Scaffold for Bioactive Compounds. Molecules.

[30] Hwang S. (2024). Antibacterial
Activity for Synthesized Coumarin Derivatives and a Coumarin Component
of Lime Peel (Citrus aurantifolia). Bioengineering.

[31] Hassan M. Z., Osman H., Ali M. A., Ahsan M. J. (2016). Therapeutic potential
of coumarins as antiviral agents. Eur. J. Med.
Chem..

[32] Yan Z., Huang Y., Zhao D., Li Z., Wang X., Guo M., Wei Y., Wang Y., Mou Y., Hou Z. (2023). Developing
Novel Coumarin-Containing Azoles Antifungal Agents by
the Scaffold Merging Strategy for Treating Azole-Resistant Candidiasis. J. Med. Chem..

[33] Bansal Y., Sethi P., Bansal G. (2013). Coumarin: a potential
nucleus for
anti-inflammatory molecules. Med. Chem. Res..

[34] Thomas V., Giles D., Basavarajaswamy G. P.
M., Das A. K., Patel A. (2017). Coumarin Derivatives as Anti-inflammatory and Anticancer Agents. Adv. Anticancer Agents Med. Chem..

[35] Abdelaziz E., El-Deeb N. M., Zayed M. F., Hasanein A. M., El Sayed I. E., Elmongy E. I., Kamoun E. A. (2023). Synthesis
and in-vitro anti-proliferative
with antimicrobial activity of new coumarin containing heterocycles
hybrids. Sci. Rep..

[36] Herrera
Herrera A., Franco Ospina L., Fang L., Díaz
Caballero A. (2014). Susceptibility of Porphyromonas gingivalis and Streptococcus
mutans to Antibacterial Effect from Mammea americana. Adv. Pharmacol. Sci..

[37] Yang H., Jiang B., Reynertson K. A., Basile M. J., Kennelly E. J. (2006). Comparative
analyses of bioactive Mammea coumarins from seven parts of Mammea
americana by HPLC-PDA with LC-MS. J. Agric.
Food Chem..

[38] Metwally N.
H., Abdallah S. O., Mohsen M. M. A. (2020). Design, green one-pot synthesis and
molecular docking study of novel N,N-bis­(cyanoacetyl)­hydrazines and
bis-coumarins as effective inhibitors of DNA gyrase and topoisomerase
IV. Bioorg. Chem..

[39] Gellert M., Mizuuchi K., O’Dea M. H., Nash H. A. (1976). DNA gyrase: an enzyme
that introduces superhelical turns into DNA. Proc. Natl. Acad. Sci. U. S. A..

[40] Maxwell A. (1993). The interaction
between coumarin drugs and DNA gyrase. Mol.
Microbiol..

[41] He Z., Jiang W., Jiang Y., Dong J., Song Z., Xu J., Zhou W. (2022). Anti-biofilm activities of coumarin as quorum sensing
inhibitor for Porphyromonas gingivalis. J. Oral
Microbiol..

[42] Campbell J. W., Cronan J. E. (2001). Bacterial fatty
acid biosynthesis: targets for antibacterial
drug discovery. Annu. Rev. Microbiol..

[43] Rana P., Ghouse S. M., Akunuri R., Madhavi Y. V., Chopra S., Nanduri S. (2020). FabI (enoyl acyl carrier
protein reductase). - A potential
broad spectrum therapeutic target and its inhibitors. Eur. J. Med. Chem..

[44] Duggirala S., Nankar R. P., Rajendran S., Doble M. (2014). Phytochemicals as inhibitors
of bacterial cell division protein FtsZ: coumarins are promising candidates. Appl. Biochem. Biotechnol..

[45] BioSolveIT BioSolveIT GmbH, SeeSAR version 13.0.1. 2023, Sankt Augustin: Germany.

[46] Berthold M. R., Cebron N., Dill F., Gabriel T. R., Kötter T., Meinl T., Ohl P., Thiel K., Wiswedel B. (2009). KNIME - the
Konstanz information miner: version 2.0 and beyond. SIGKDD Explor. Newsl.

[47] Heidemann J. L., Neumann P., Dickmanns A., Ficner R. (2019). Crystal structures
of the c-di-AMP-synthesizing enzyme CdaA. J.
Biol. Chem..

[48] Liu C., Worthington R. J., Melander C., Wu H. (2011). A New Small Molecule
Specifically Inhibits the Cariogenic Bacterium Streptococcus mutans
in Multispecies Biofilms. Antimicrob. Agents
Chemother.

[49] GraphPad Software GraphPad Prism Version 10.3.0 for Windows. GraphPad Software 2024.

[50] Mota F. L., Queimada A. J., Pinho S. P., Macedo E. A. (2008). Aqueous Solubility
of Some Natural Phenolic Compounds. Ind. Engg.
Chem. Res..

[51] Stieger N., Liebenberg W., Wessels J. C. (2009). UV Spectrophotometric Method for
the Identification and Solubility Determination of Nevirapine. Pharmazie.

[52] Wang Y., Wang J., Fu Z., Sheng R., Wu W., Fan J., Guo R. (2020). Syntheses
and evaluation of daphnetin derivatives as
novel G protein-coupled receptor inhibitors and activators. Bioorg. Chem..

