# Supplementary material for: Coumarin-Mediated Inhibition of Diadenylate Cyclase Correlates with Impaired Biofilm Formation in Streptococcus mutans
Source: ACS Infect Dis. 2026 Jul 1;12(7):2188–202. doi: 10.1021/acsinfecdis.5c00767 (PMC13366584; doi:10.1021/acsinfecdis.5c00767)
Supplement: Supplementary file 1 [file id5c00767_si_001.pdf]

Supporting information

Coumarin-mediated inhibition of diadenylate cyclase correlates with impaired biofilm formation in *Streptococcus mutans*

Edwin M. Rojas[1,2, ‡] *Abhishek Govindan*[1, ‡], *Parvathy Babu*[1], *Soniya Joseph*[1], *Hua Zhang*[5], *Yanting Zhu*[5], *Tyrese Boddie*[1], *Hui-Ting Lee*[1], *Hui Wu*[5]*, *and Sadanandan E. Velu*[1,3,4]*

[1]*Department of Chemistry,* [2]*School of Dentistry,* [3]*Global Center for Craniofacial Oral and Dental Disorders,* [4]*Center for Clinical and Translational Sciences, University of Alabama at Birmingham, Birmingham, AL 35294, USA*
[5]*Division of Biomaterial and Biomedical Sciences, School of Dentistry, Oregon Health & Science University, Portland, OR 97239, USA*

Running title: Coumarins inhibit smDAC and biofilms.

Keywords: S. mutans, smDAC, biofilm, c-di-AMP, coumarin

‡ Co-first authors

* Corresponding authors:
Sadanandan E. Velu, PhD, Phone: (205) 975-2478, Email: svelu@uab.edu
Hui Wu, PhD, Phone: 503-418-2090, Email: wuhu@ohsu.edu

Table of Contents

1. Copies of ^{1}H NMR and ^{13}C NMR Spectra................................ .... S2
2. HPLC Chromatograms S13

^{1}H and ^{13}C spectra of coumarins

Figure S1: 3-Benzyl-7,8-dihydroxy-4-methyl-2H-chromen-2-one (6)

^{1}H NMR (400 MHz, DMSO-d_6)

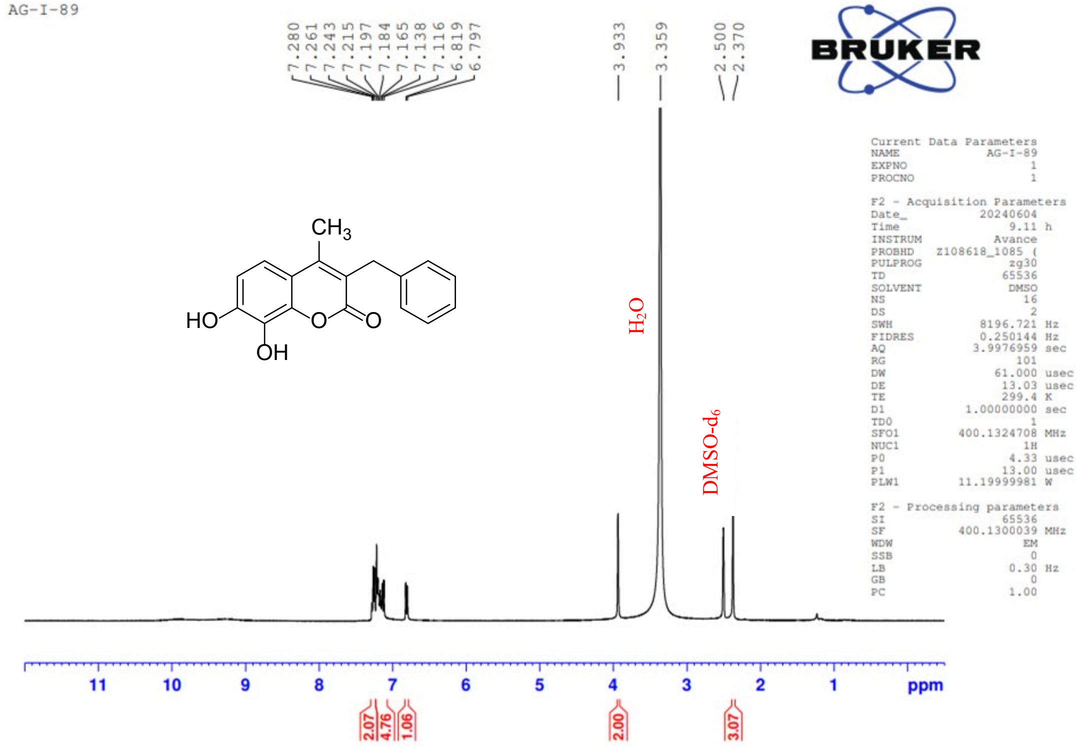

Figure S2: 3-Benzyl-7,8-dihydroxy-4-methyl-2H-chromen-2-one (6)

^{13}C NMR (100 MHz, DMSO-d_6)

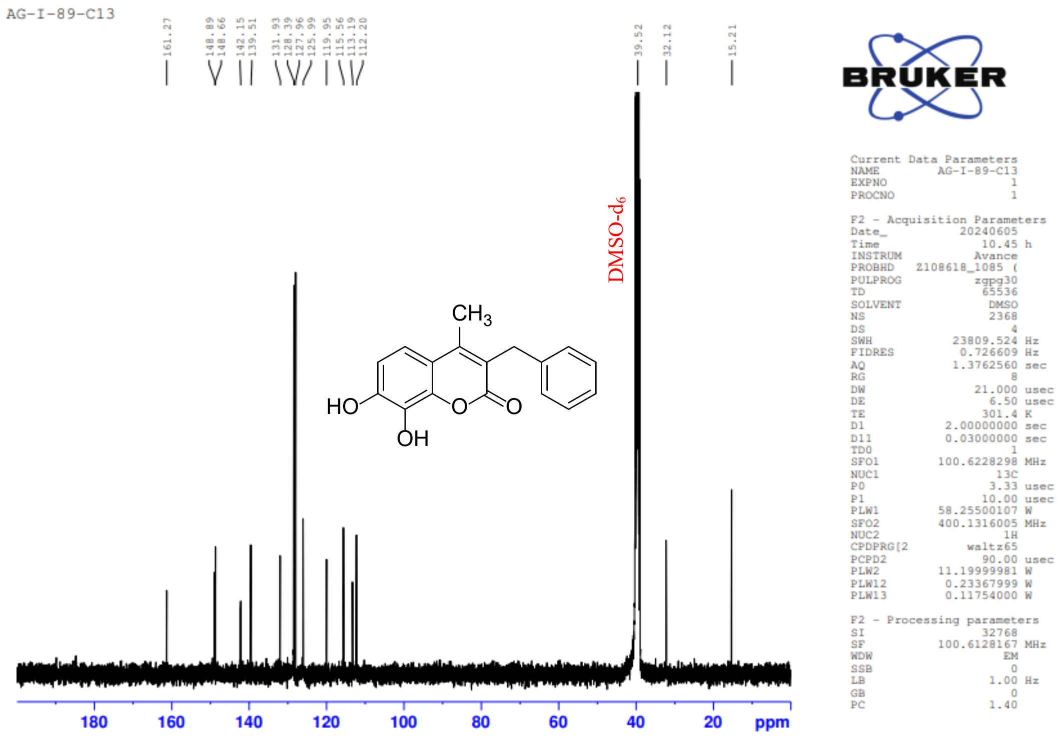

Figure S3: 7,8-Dihydroxy-4-methyl-3-(3-(trifluoromethyl)benzyl)-2H-chromen-2-one (17)
^{1}H NMR (400 MHz, DMSO-d_6)

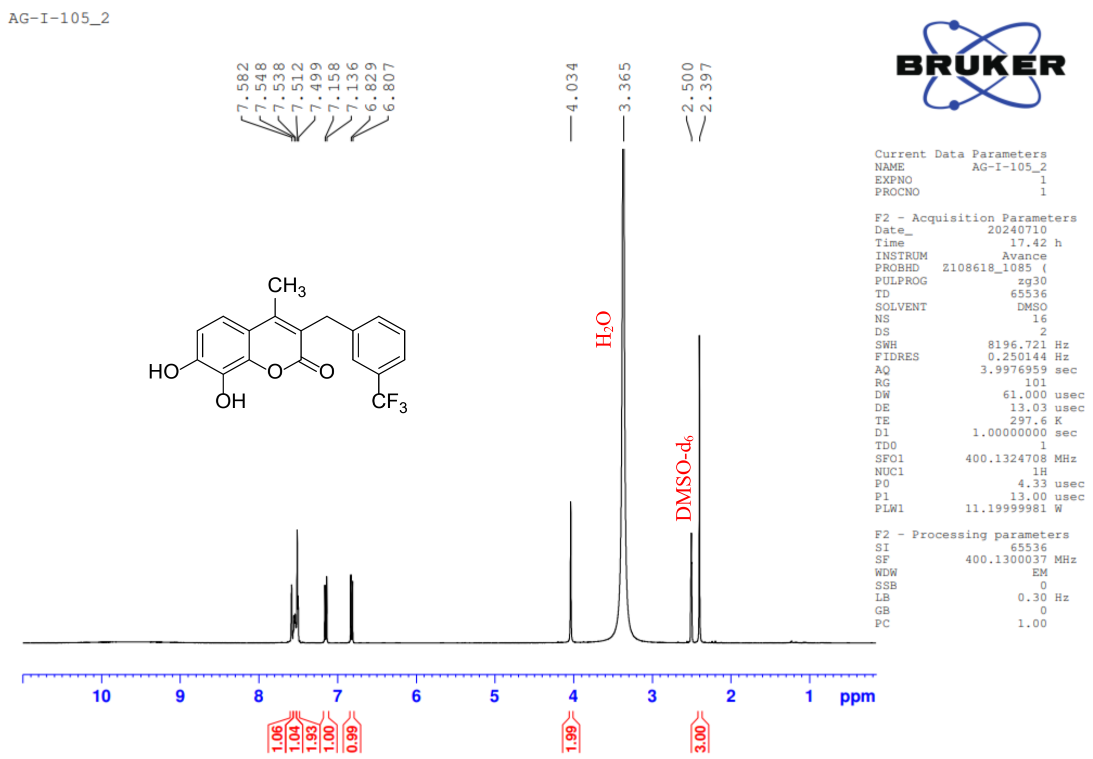

Figure S4: 7,8-Dihydroxy-4-methyl-3-(3-(trifluoromethyl)benzyl)-2H-chromen-2-one (17)
^{13}C NMR (100 MHz, DMSO-d_6)

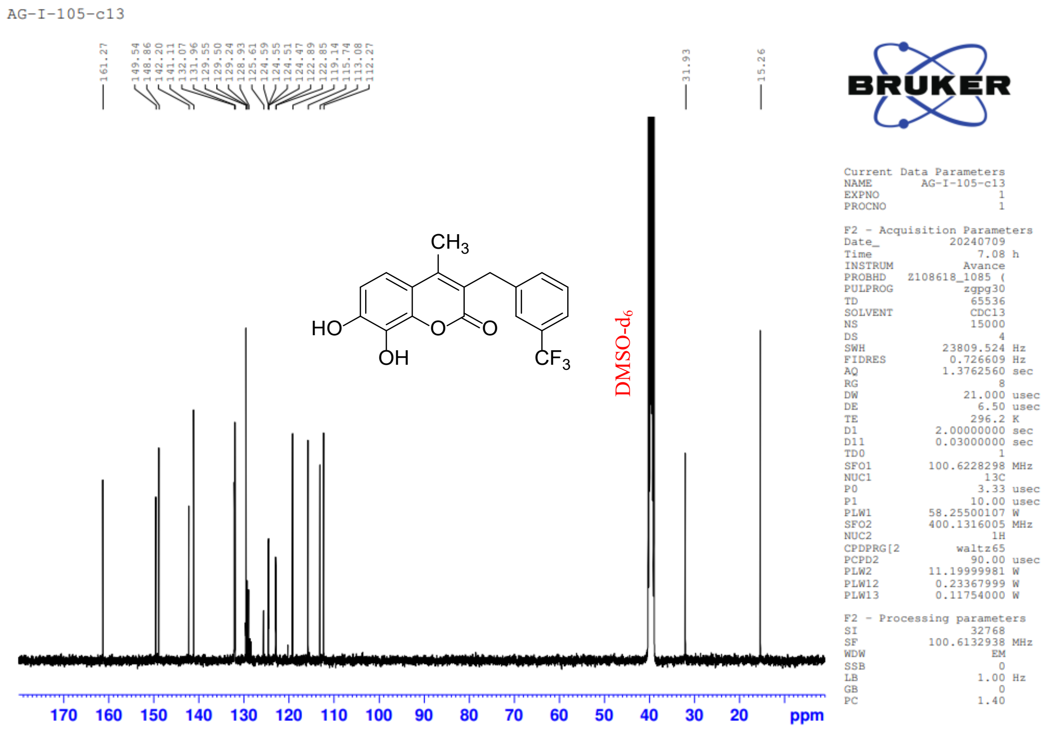

Figure S5: 7,8-Dihydroxy-4-methyl-3-(4-(trifluoromethyl)benzyl)-2H-chromen-2-one (18)

^{1}H NMR (400 MHz, DMSO-d_6)

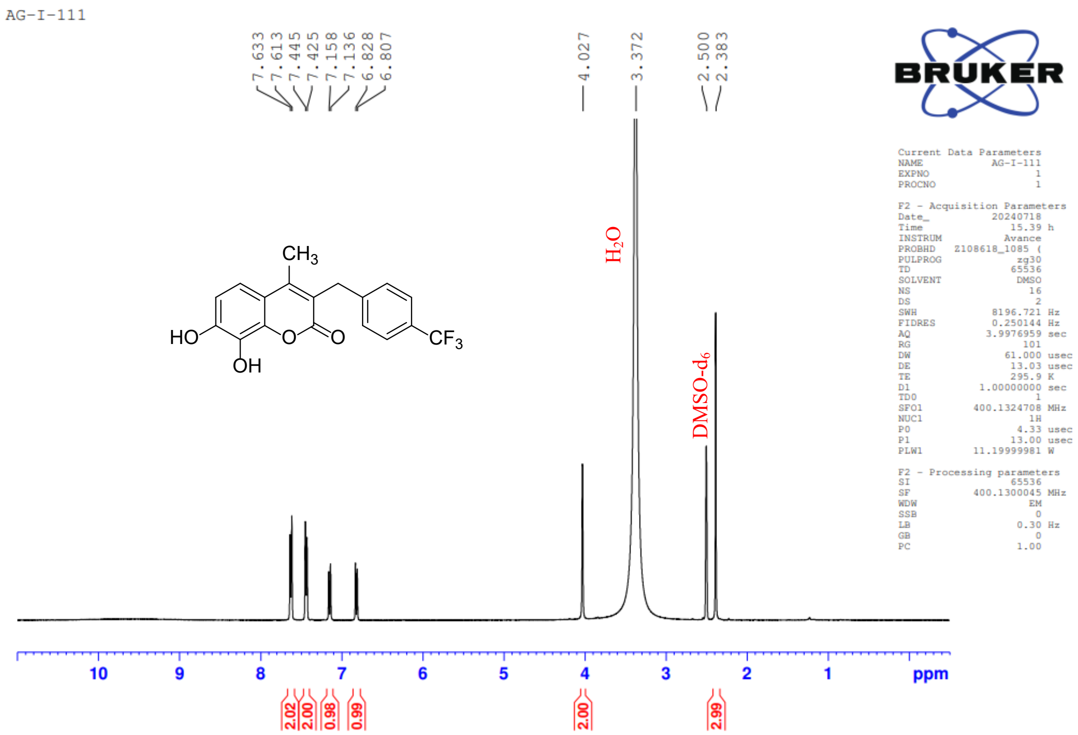

Figure S6: 7,8-Dihydroxy-4-methyl-3-(4-(trifluoromethyl)benzyl)-2H-chromen-2-one (18)

^{13}C NMR (100 MHz, DMSO-d_6)

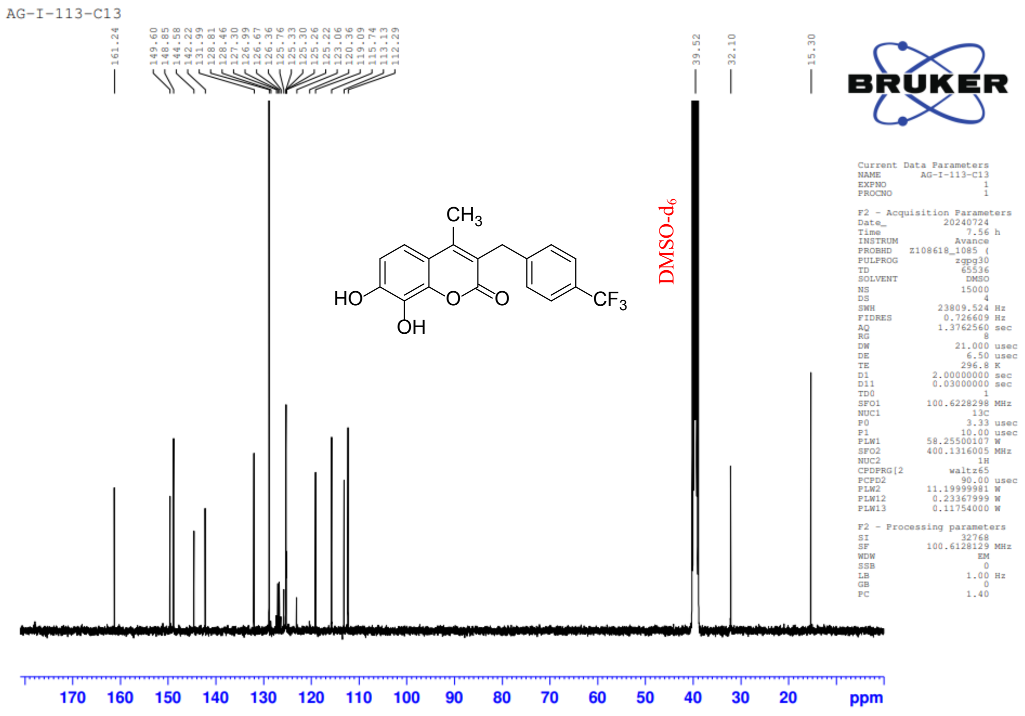

Figure S7: 3-(4-Chlorobenzyl)-7,8-dihydroxy-4-methyl-2H-chromen-2-one (19)

^{1}H NMR (400 MHz, DMSO-d_6)

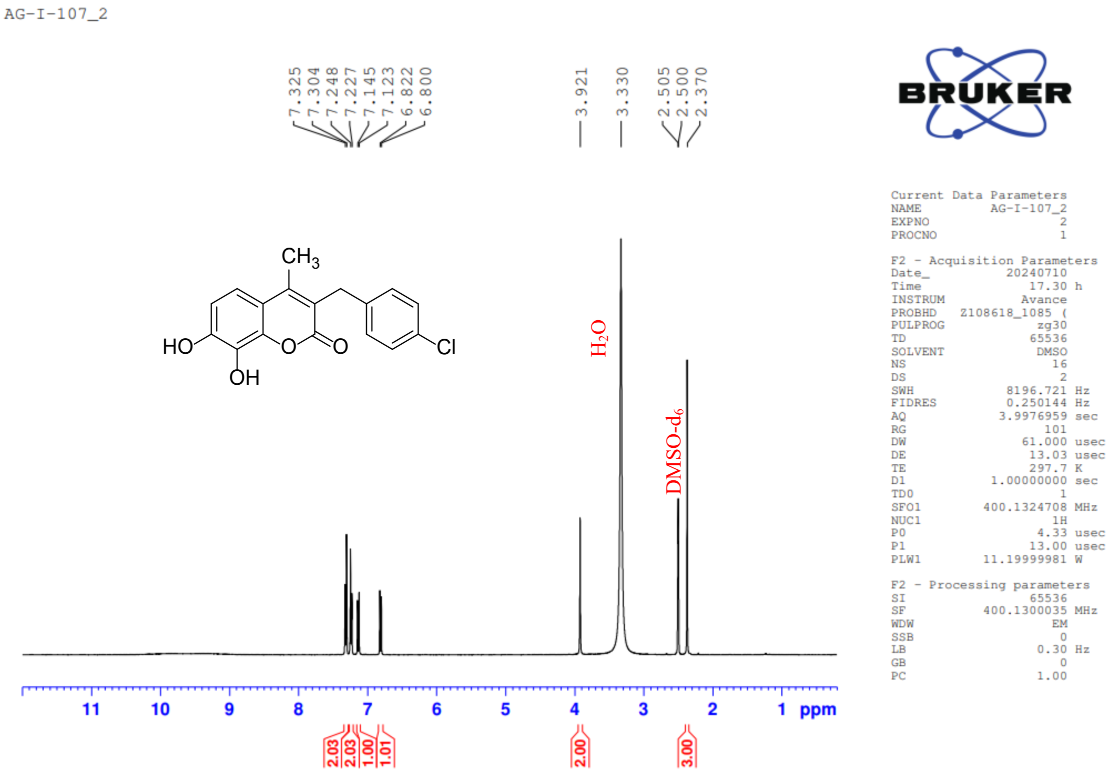

Figure S8: 3-(4-Chlorobenzyl)-7,8-dihydroxy-4-methyl-2H-chromen-2-one (19)

^{13}C NMR (100 MHz, DMSO-d_6)

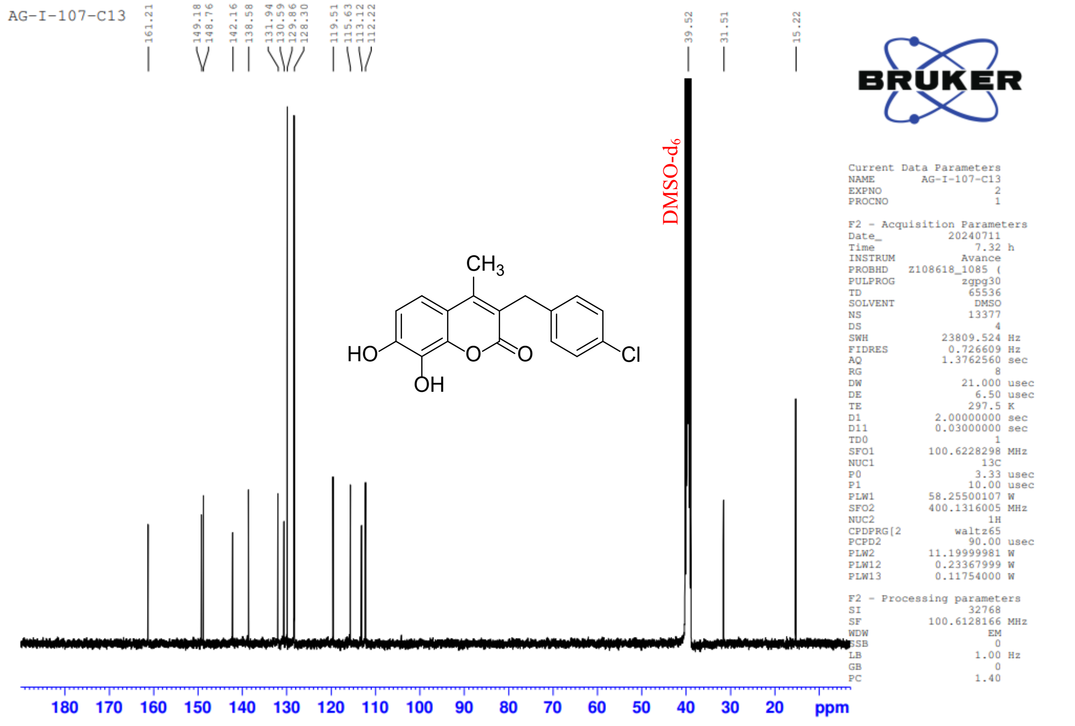

Figure S9: 3-(4-Fluorobenzyl)-7,8-dihydroxy-4-methyl-2H-chromen-2-one (20)
^{1}H NMR (400 MHz, DMSO-d_6)

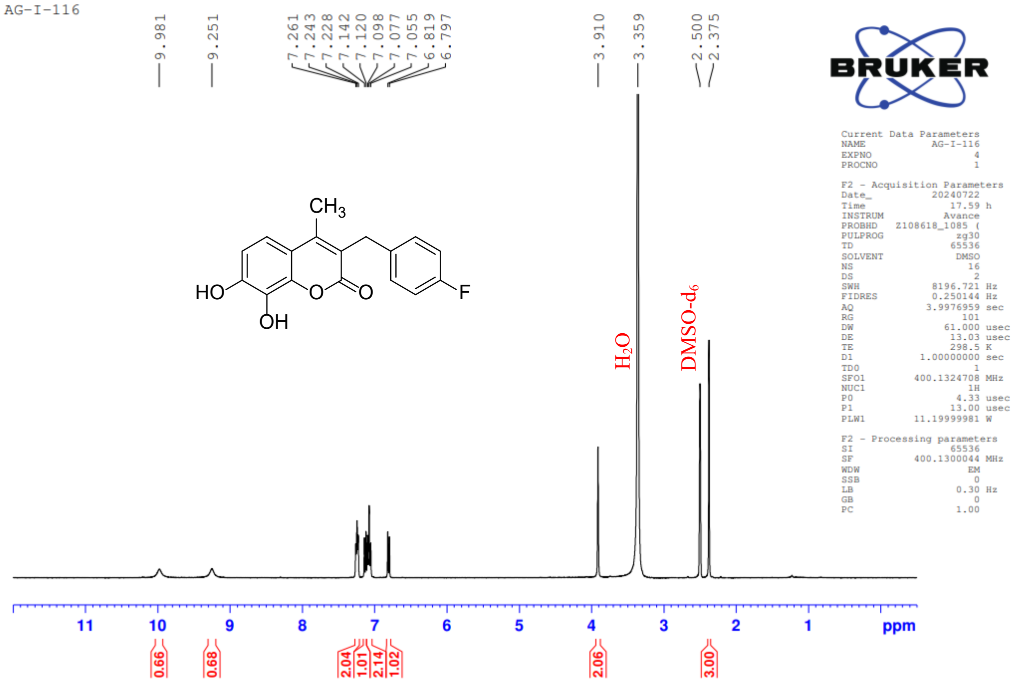

Figure S10: 3-(4-Fluorobenzyl)-7,8-dihydroxy-4-methyl-2H-chromen-2-one (20)
^{13}C NMR (100 MHz, DMSO-d_6)

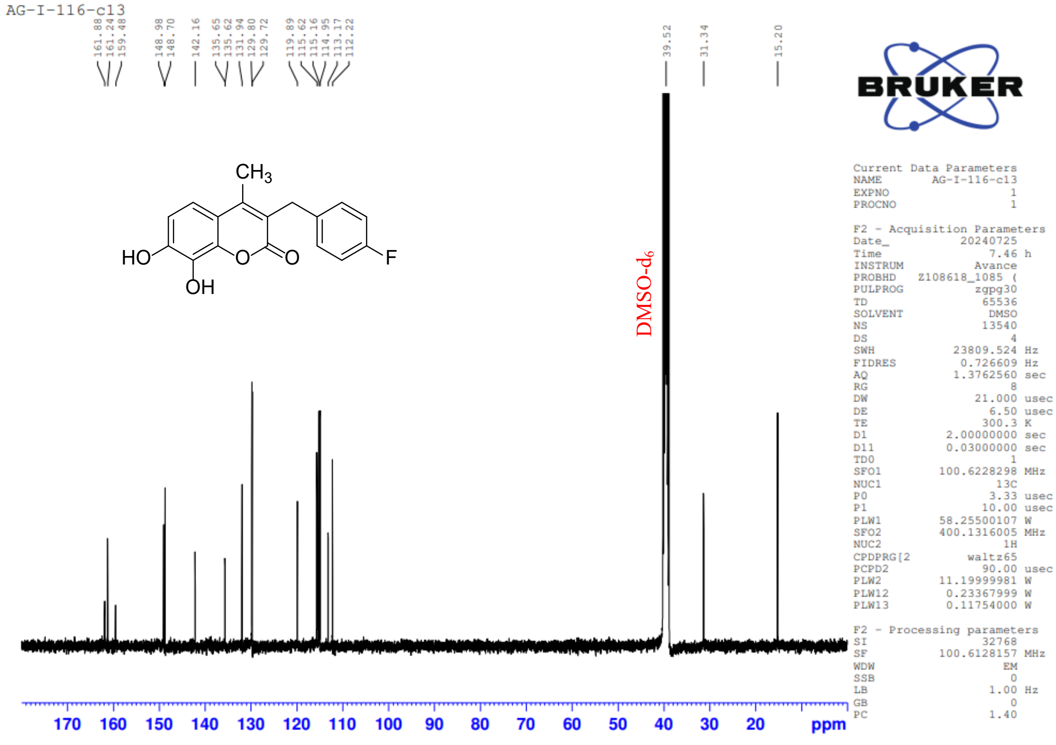

Figure S11: 7,8-Dihydroxy-4-methyl-3-(3-methylbenzyl)-2H-chromen-2-one (21)
^{1}H NMR (400 MHz, DMSO-d_6)

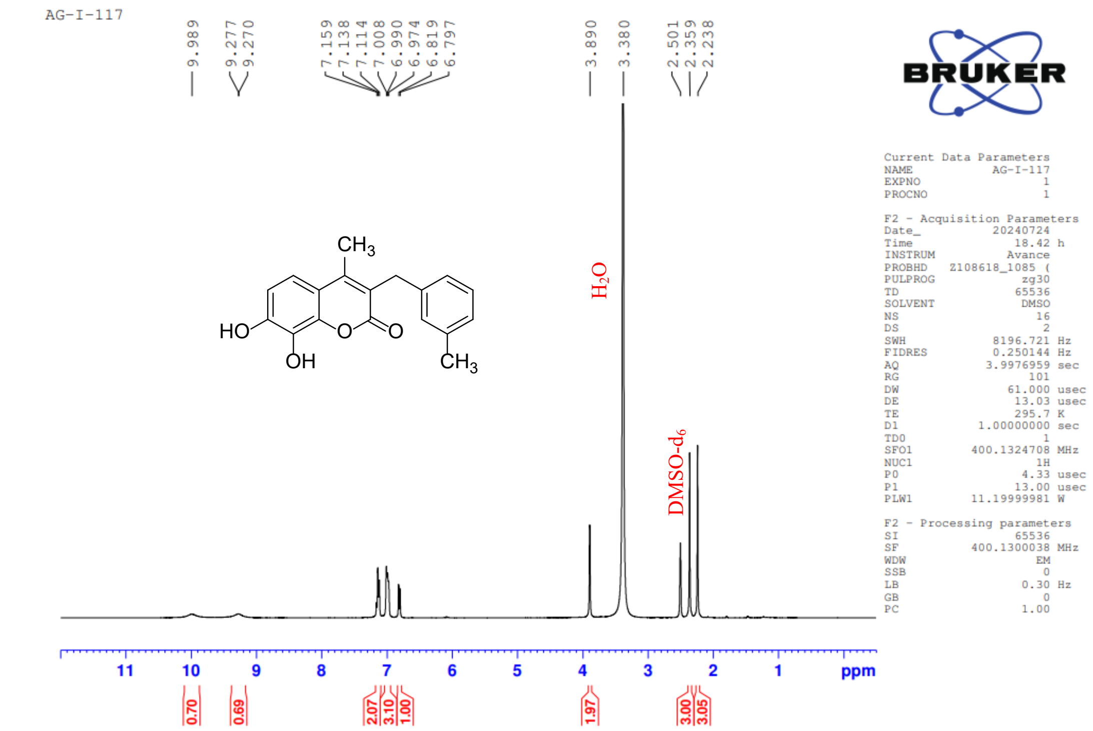

Figure S12: 7,8-Dihydroxy-4-methyl-3-(3-methylbenzyl)-2H-chromen-2-one (21)
^{13}C NMR (100 MHz, DMSO-d_6)

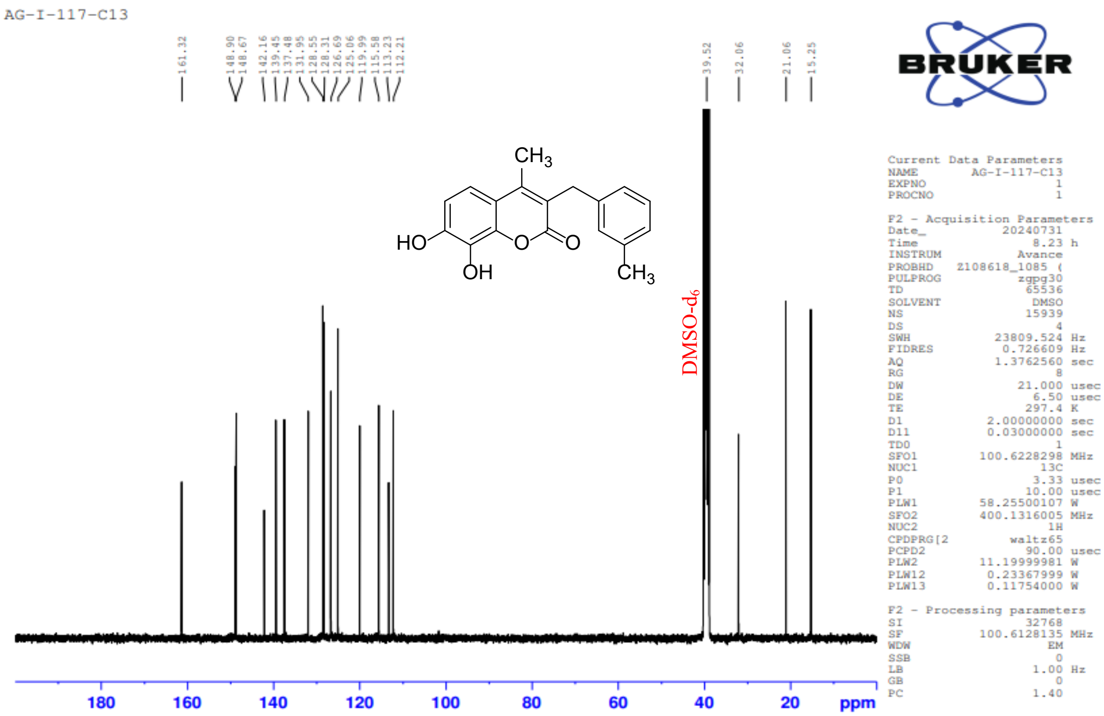

Figure S13: 7,8-Dihydroxy-4-methyl-2H-chromen-2-one (22)
^{1}H NMR (400 MHz, DMSO-d_6)

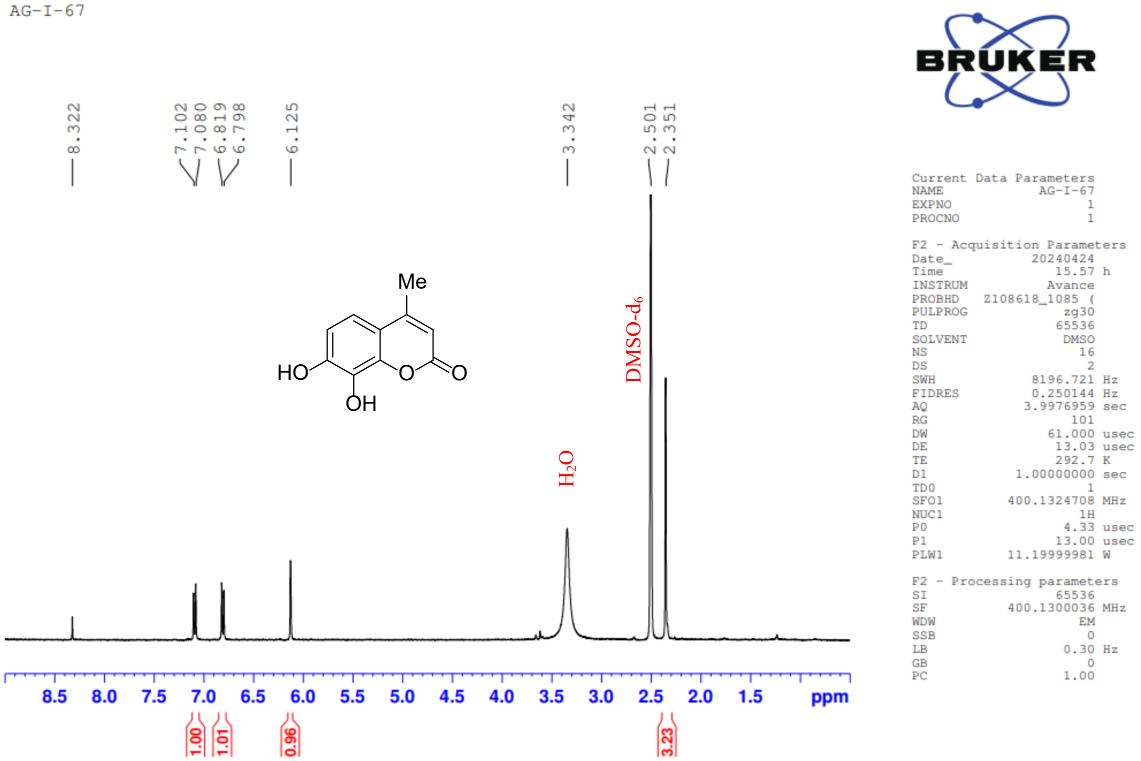

Figure S14: 7,8-Dihydroxy-4-methyl-2H-chromen-2-one (22)
^{13}C NMR (100 MHz, DMSO-d_6)

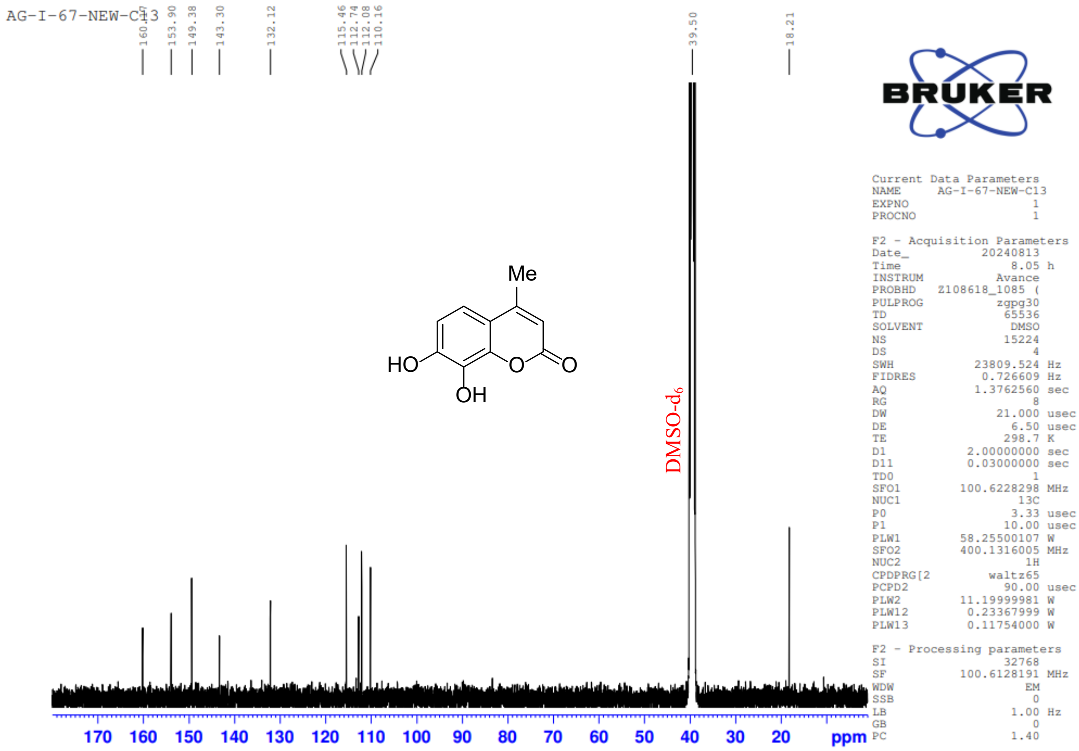

Figure S15: 7,8-Dihydroxy-2-oxo-2H-chromene-3-carboxylic acid (23)
^{1}H NMR (400 MHz, DMSO-d_6)

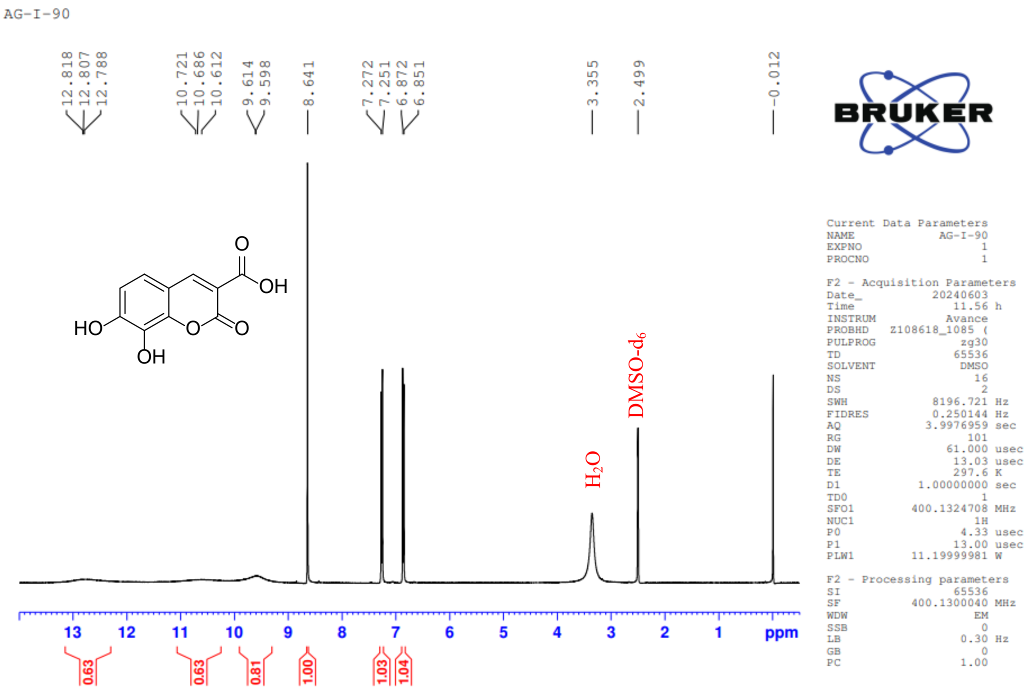

Figure S16: 7,8-Dihydroxy-2-oxo-2H-chromene-3-carboxylic acid (23)
^{13}C NMR (100 MHz, DMSO-d_6)

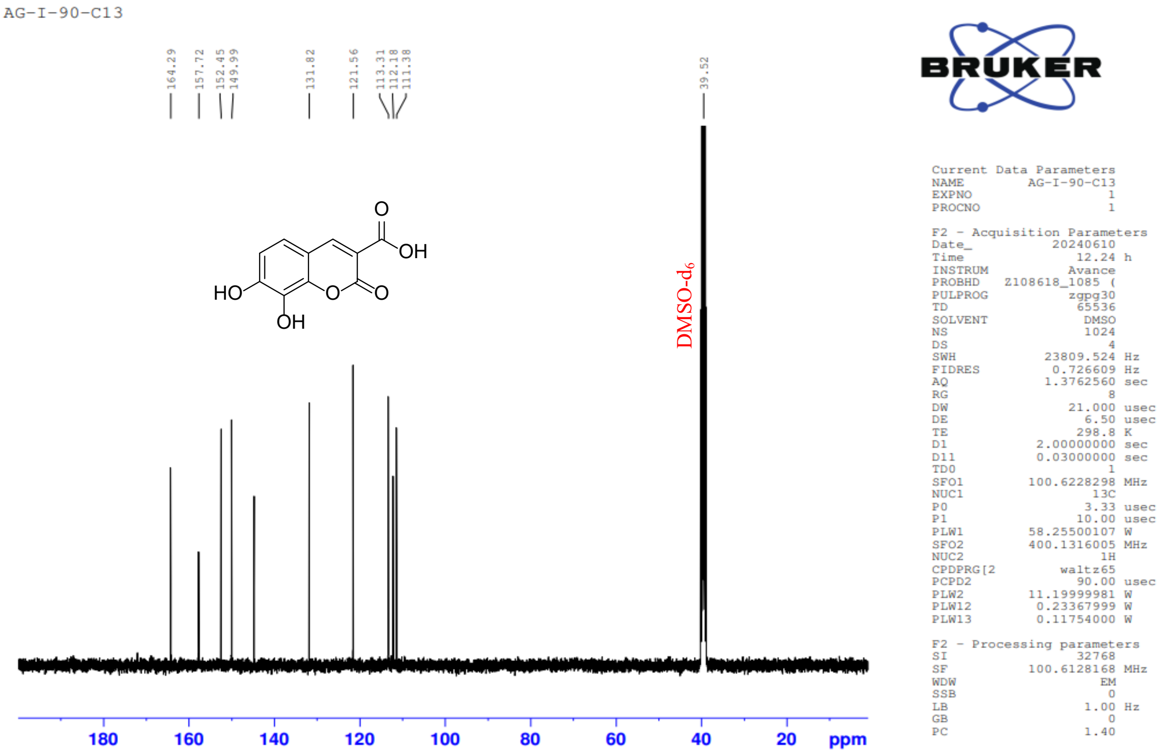

Figure S17: Methyl 7,8-dihydroxy-2-oxo-2H-chromene-3-carboxylate (24)

^{1}H NMR (400 MHz, DMSO-d_6)

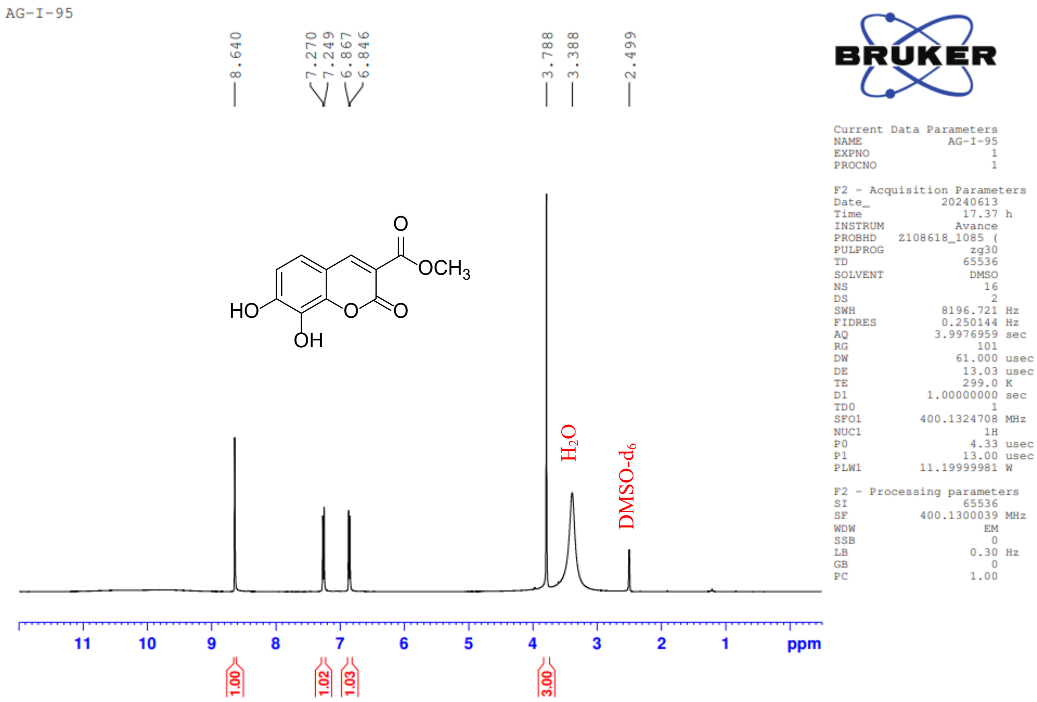

Figure S18: Methyl 7,8-dihydroxy-2-oxo-2H-chromene-3-carboxylate (24)

^{13}C NMR (100 MHz, DMSO-d_6)

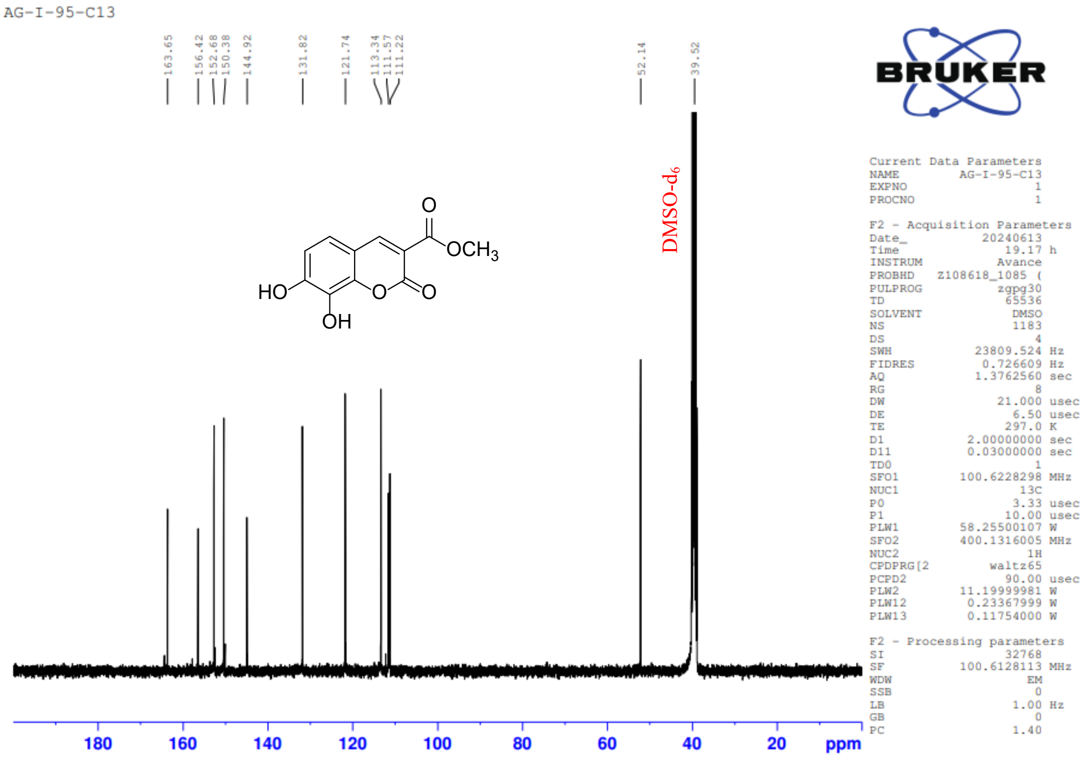

Figure S19: 7,8-Dimethoxy-4-methyl-2H-chromen-2-one (25)
^{1}H NMR (400 MHz, DMSO-d_6)

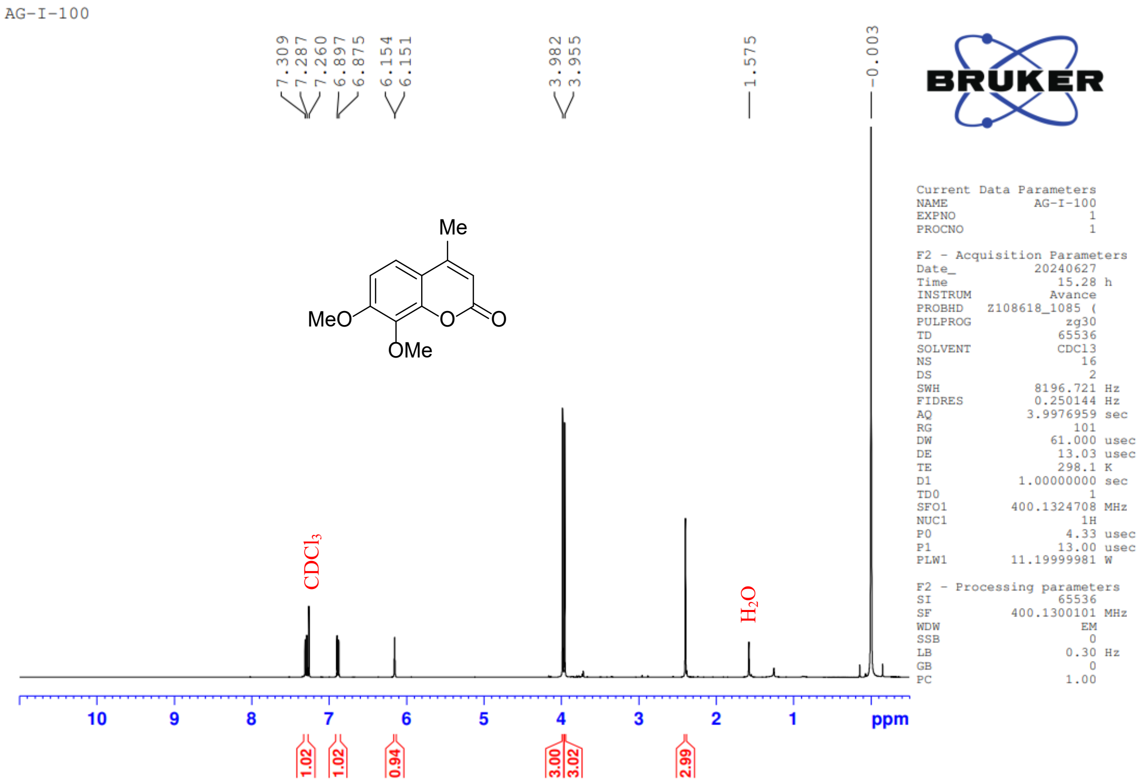

Figure S20: 7,8-Dimethoxy-4-methyl-2H-chromen-2-one (25)
^{13}C NMR (100 MHz, DMSO-d_6)

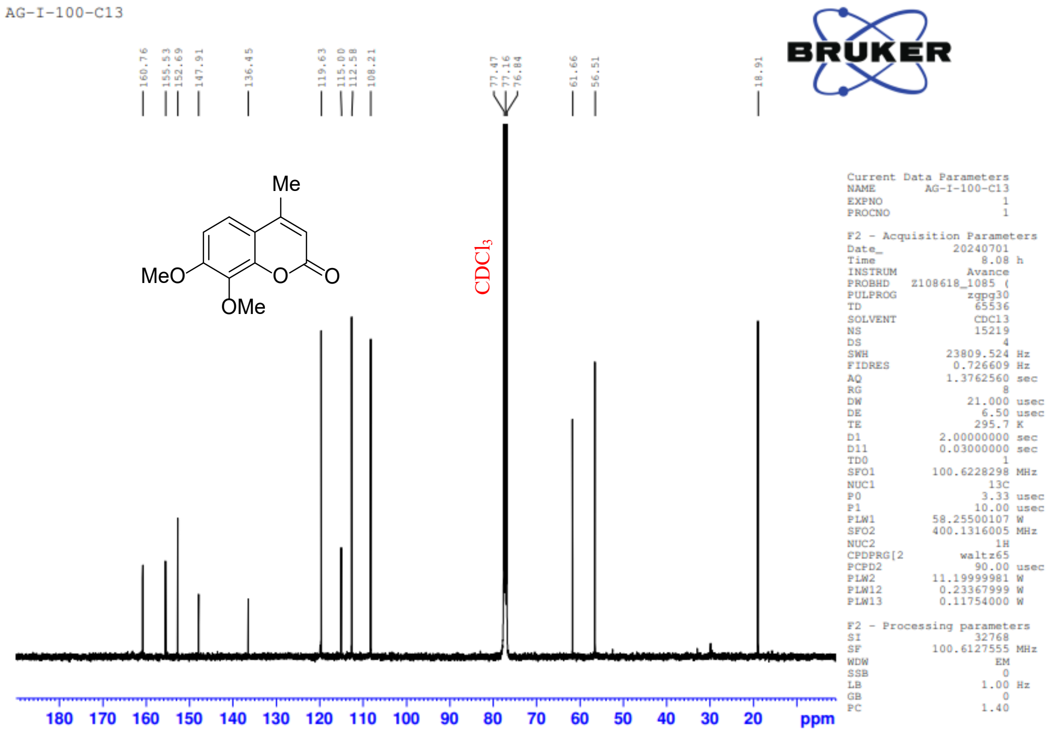

Figure S21: Benzene-1,2,3-triyl triacetate (29)

^{1}H NMR (300 MHz, $CDCl_3$)

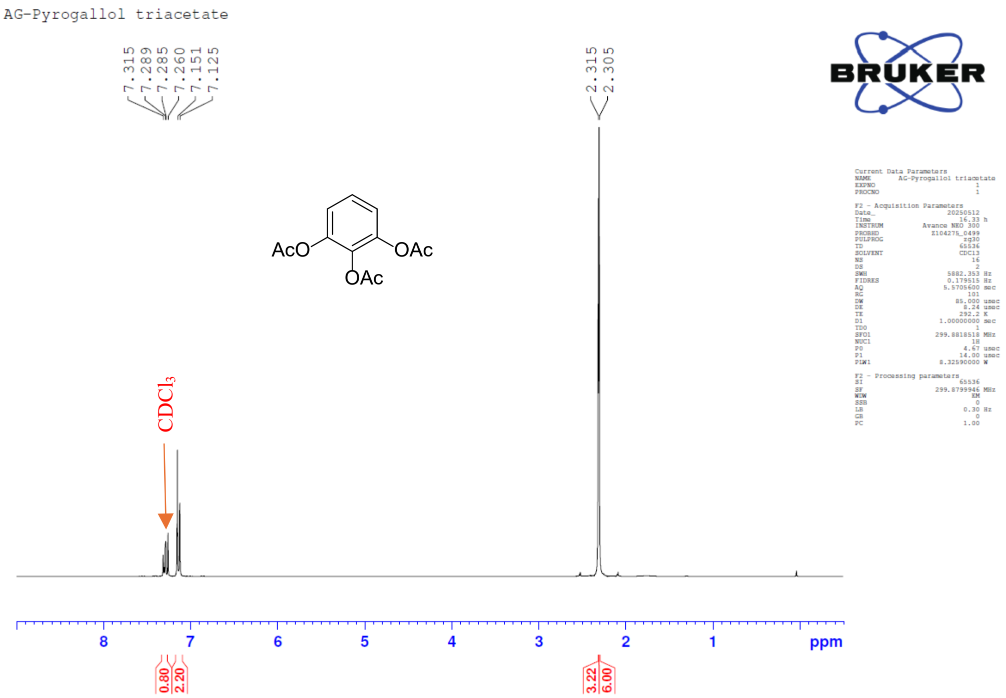

Figure S22: Benzene-1,2,3-triyl triacetate (29)

^{13}C NMR (75 MHz, $CDCl_3$)

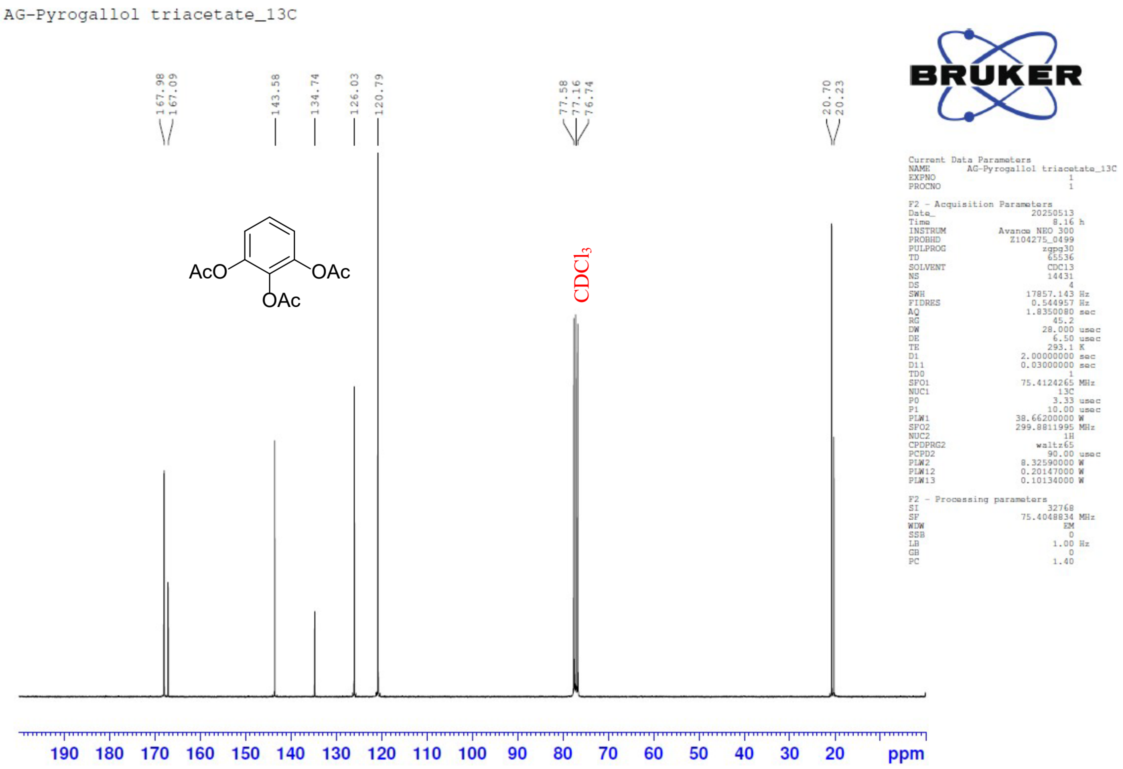

HPLC analysis of coumarins

Buffer 75:25, MeCN, H_2O

Figure S23: HPLC grade MeOH - Control

<Chromatogram>

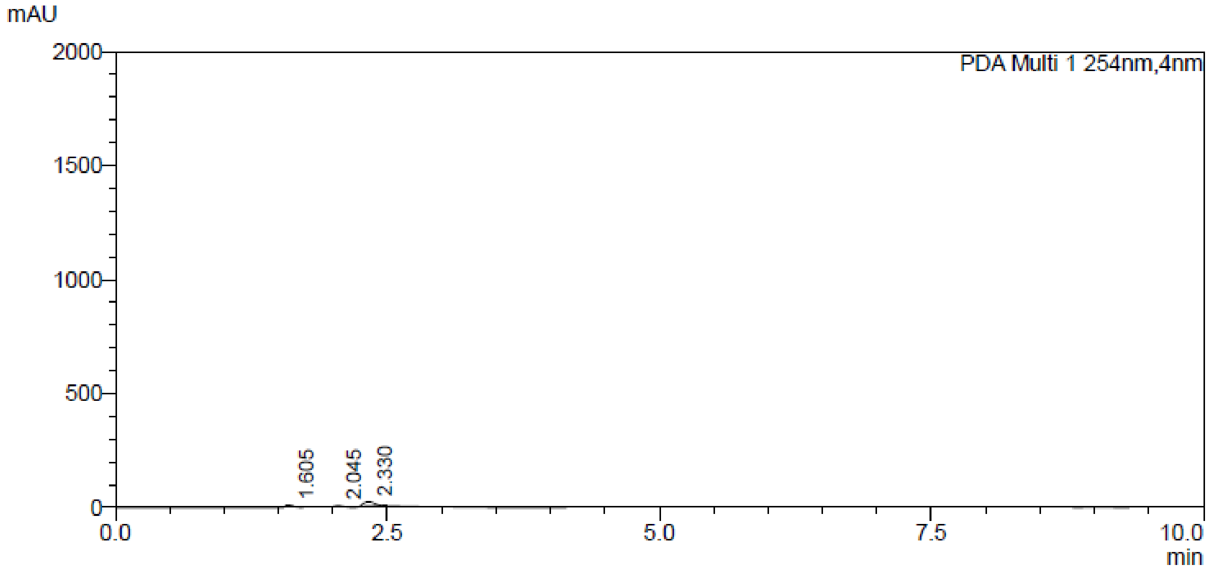

<Peak Table>

PDA Ch1 254nm

Peak#	Ret. Time	Area	Height	Area%
1	1.605	70156	14106	29.589
2	2.045	42352	8448	17.863
3	2.330	124589	18707	52.548
Total		237098	41261	100.000

Figure S24: 3-Benzyl-7,8-dihydroxy-4-methyl-2H-chromen-2-one (6)

<Chromatogram>

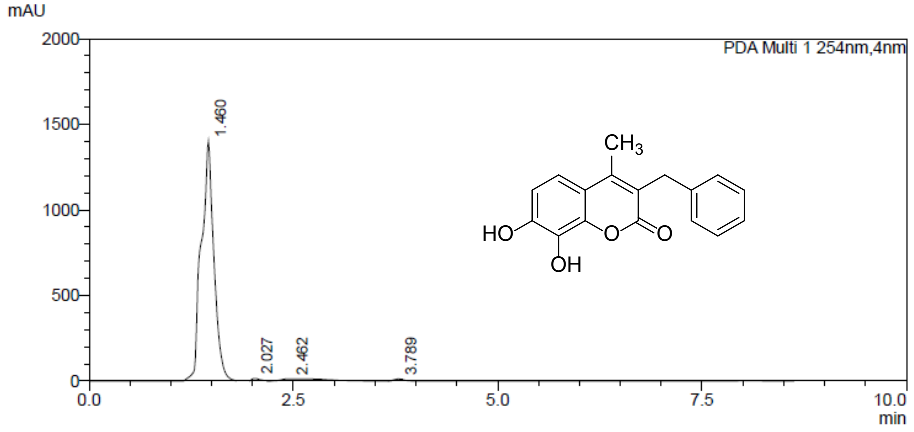

<Peak Table>

PDA Ch1 254nm

Peak#	Ret. Time	Area	Height	Area%
1	1.460	15128464	1404529	96.297
2	2.027	148677	18453	0.946
3	2.462	404822	11769	2.577
4	3.789	28320	4877	0.180
Total		15710282	1439628	100.000

Figure S25: 7,8-Dihydroxy-4-methyl-3-(3-(trifluoromethyl)benzyl)-2H-chromen-2-one (17)

<Chromatogram>

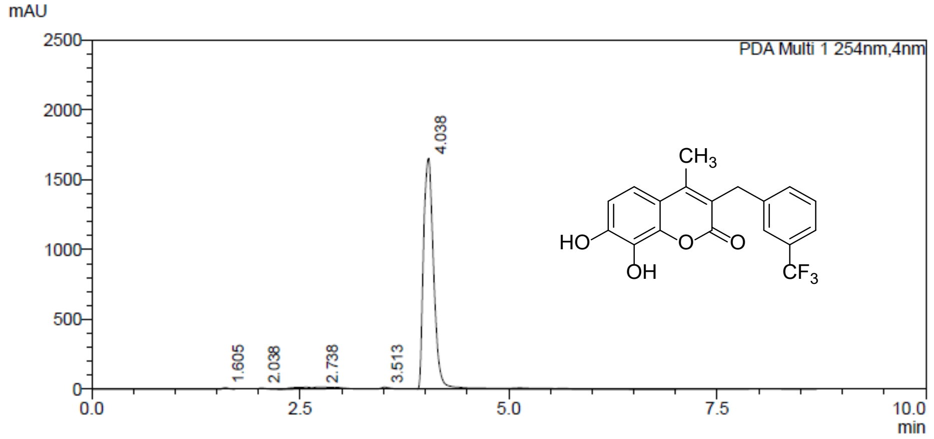

<Peak Table>

PDA Ch1 254nm

Peak#	Ret. Time	Area	Height	Area%
1	1.605	58345	11215	0.387
2	2.038	62879	10327	0.417
3	2.738	446724	13075	2.966
4	3.513	98153	13002	0.652
5	4.038	14395376	1648665	95.577
Total		15061478	1696285	100.000

Figure S26: 7,8-Dihydroxy-4-methyl-3-(4-(trifluoromethyl)benzyl)-2H-chromen-2-one (18)

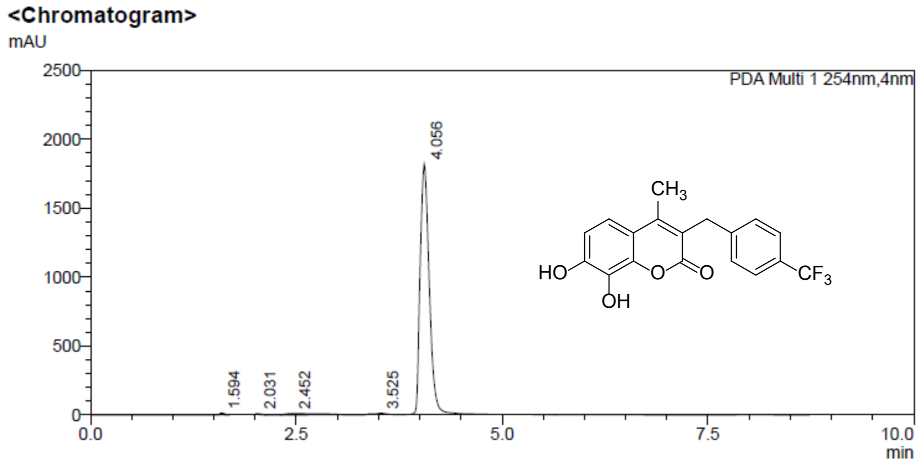

<Peak Table>

PDA Ch1 254nm

Peak#	Ret. Time	Area	Height	Area%
1	1.594	52505	11918	0.359
2	2.031	29714	6640	0.203
3	2.452	340596	11605	2.331
4	3.525	87846	9209	0.601
5	4.056	14101914	1814393	96.505
Total		14612576	1853765	100.000

Figure S27: 3-(4-Chlorobenzyl)-7,8-dihydroxy-4-methyl-2H-chromen-2-one (19)

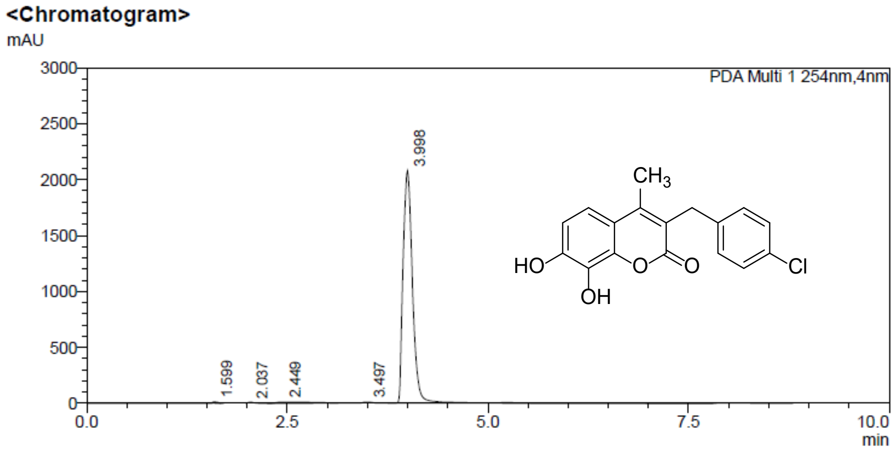

<Peak Table>

PDA Ch1 254nm

Peak#	Ret. Time	Area	Height	Area%
1	1.599	49546	11602	0.276
2	2.037	75561	12052	0.422
3	2.449	382098	12572	2.132
4	3.497	74731	10091	0.417
5	3.998	17341107	2076194	96.753
Total		17923043	2122511	100.000

Figure S28: 3-(4-Fluorobenzyl)-7,8-dihydroxy-4methyl-2H-chromen-2-one (20)

<Chromatogram>

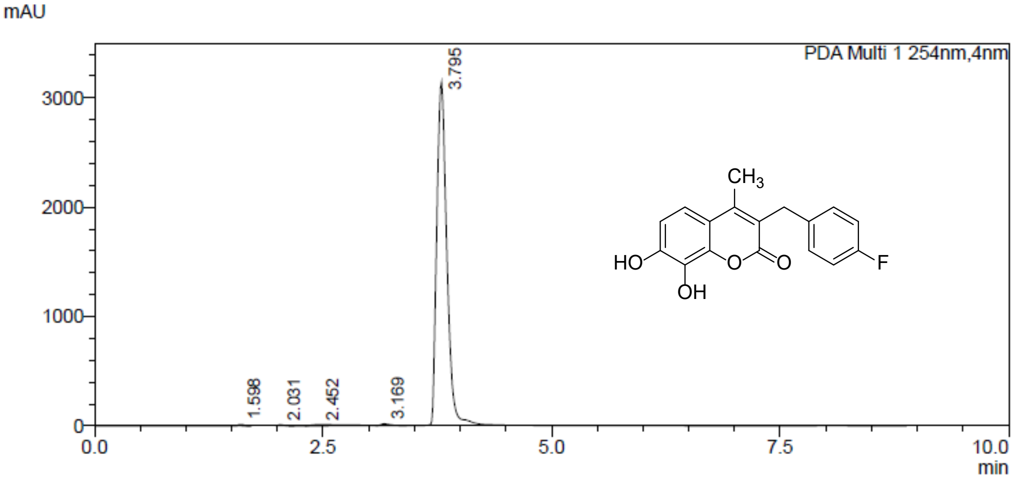

<Peak Table>

PDA Ch1 254nm

Peak#	Ret. Time	Area	Height	Area%
1	1.598	56724	13046	0.228
2	2.031	29428	6763	0.118
3	2.452	282725	10889	1.138
4	3.169	100268	14962	0.404
5	3.795	24368414	3143093	98.111
Total		24837560	3188754	100.000

Figure S29: 7,8-Dihydroxy-4-methyl-3-(3-methylbenzyl)-2H-chromen-2-one (21)

<Chromatogram>

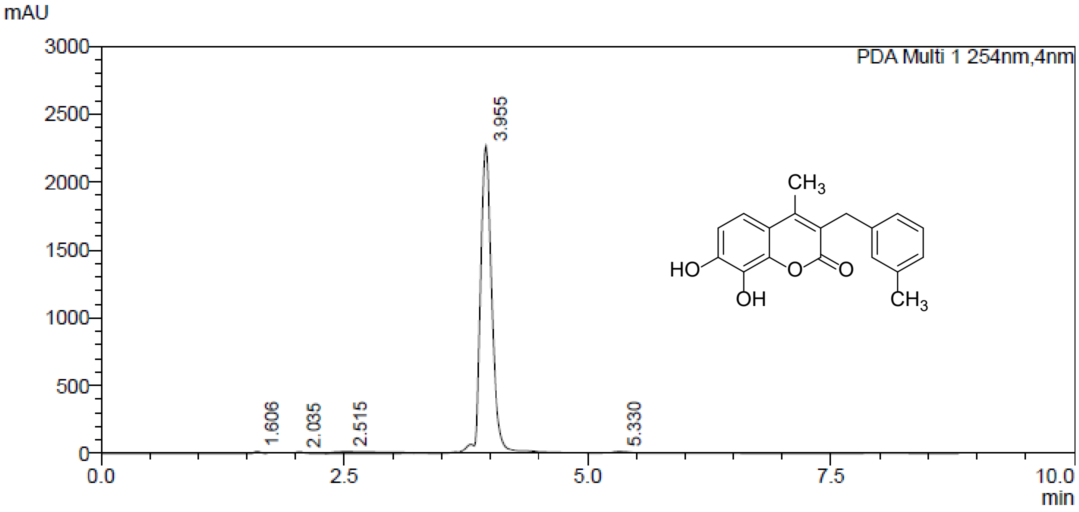

<Peak Table>

PDA Ch1 254nm

Peak#	Ret. Time	Area	Height	Area%
1	1.606	80263	15954	0.414
2	2.035	76132	12131	0.393
3	2.515	488124	13686	2.520
4	3.955	18678062	2264342	96.444
5	5.330	44068	6583	0.228
Total		19366649	2312696	100.000

Figure S30: 7,8-Dihydroxy-4-methyl-2H-chromen-2-one (22)

<Chromatogram>

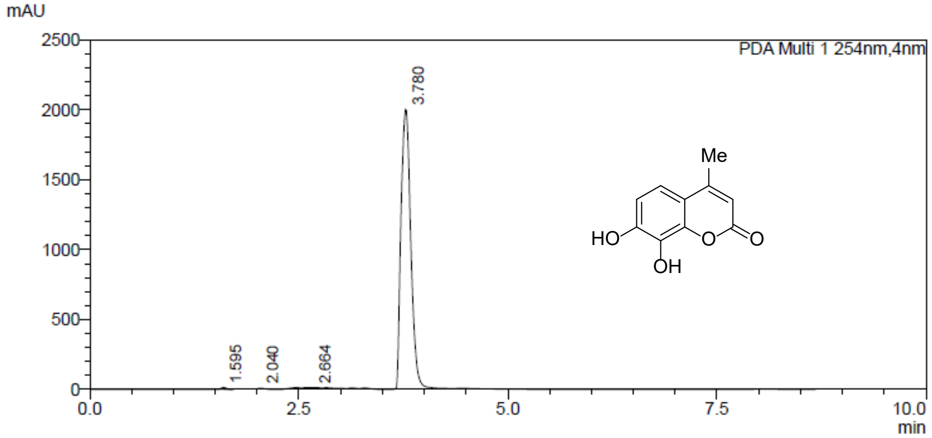

<Peak Table>

PDA Ch1 254nm

Peak#	Ret. Time	Area	Height	Area%
1	1.595	60008	13254	0.348
2	2.040	56280	10948	0.327
3	2.664	353604	8459	2.052
4	3.780	16763586	2001446	97.273
Total		17233479	2034107	100.000

Figure S31: 7,8-Dihydroxy-2-oxo-2H-chromene-3-carboxylic acid (23)

<Chromatogram>

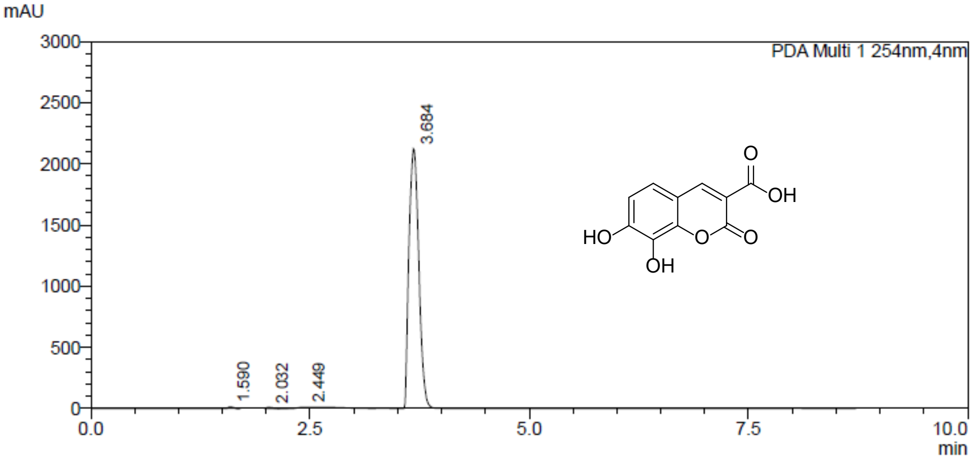

<Peak Table>

PDA Ch1 254nm

Peak#	Ret. Time	Area	Height	Area%
1	1.590	48984	11662	0.287
2	2.032	41464	10311	0.243
3	2.449	215037	8701	1.262
4	3.684	16737279	2123191	98.208
Total		17042764	2153865	100.000

Figure S32: Methyl 7,8-dihydroxy-2-oxo-2H-chromene-3-carboxylate (24)

<Chromatogram>

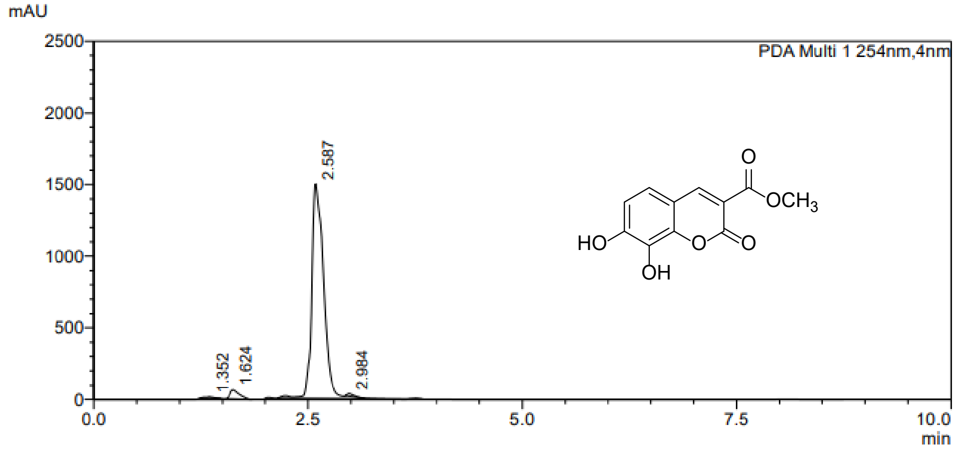

<Peak Table>

PDA Ch1 254nm

Peak#	Ret. Time	Area	Height	Area%
1	1.352	68214	9620	0.455
2	1.624	480433	63245	3.208
3	2.587	14335193	1494176	95.718
4	2.984	92593	18618	0.618
Total		14976434	1585659	100.000

Figure S33: 7,8-Dimethoxy-4-methyl-2H-chromen-2-one (25)

<Chromatogram>

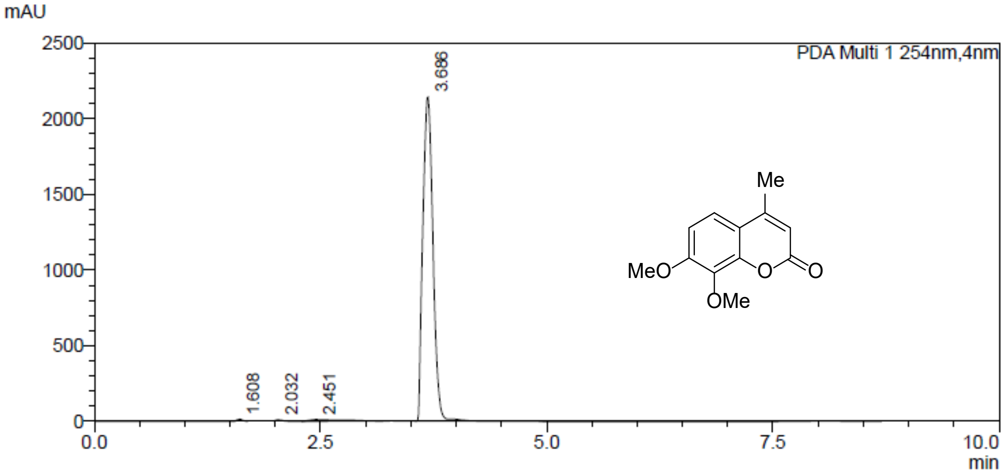

<Peak Table>

PDA Ch1 254nm

Peak#	Ret. Time	Area	Height	Area%
1	1.608	52120	12793	0.303
2	2.032	62734	10898	0.365
3	2.451	365275	12385	2.124
4	3.686	16719250	2140900	97.208
Total		17199379	2176975	100.000
